# How Breast Cancer Patients Want to Search for and Retrieve Information From Stories of Other Patients on the Internet: an Online Randomized Controlled Experiment

**DOI:** 10.2196/jmir.1215

**Published:** 2010-03-09

**Authors:** Regina Overberg, Wilma Otten, Andries de Man, Pieter Toussaint, Judith Westenbrink, Bertie Zwetsloot-Schonk

**Affiliations:** ^4^The Amazones FoundationHaarlemThe Netherlands; ^3^Department of Computer and Information ScienceNorwegian University of Science and TechnologyTrondheimNorway; ^2^Medical Decision MakingLeiden University Medical CenterLeidenThe Netherlands; ^1^Clinical Informatics GroupLeiden University Medical CenterLeidenThe Netherlands

**Keywords:** Breast cancer, life experiences, social support, Internet, information retrieval, patient satisfaction.

## Abstract

**Background:**

Other patients’ stories on the Internet can give patients information, support, reassurance, and practical advice.

**Objectives:**

We examined which search facility for online stories resulted in patients’ satisfaction and search success.

**Methods:**

This study was a randomized controlled experiment with a 2x2 factorial design conducted online. We facilitated access to 170 stories of breast cancer patients in four ways based on two factors: (1) no versus yes search by story topic, and (2) no versus yes search by writer profile. Dutch speaking women with breast cancer were recruited. Women who gave informed consent were randomly assigned to one of four groups. After searching for stories, women were offered a questionnaire relating to satisfaction with the search facility, the stories retrieved, and impact of the stories on coping with breast cancer. Of 353 enrolled women, 182 (51.6%) completed the questionnaire: control group (n = 37), story topics group (n = 49), writer profile group (n = 51), and combination group (n = 45).

**Results:**

Questionnaire completers were evenly distributed over the four groups (χ^2^_3_ = 3.7, *P* = .30). Women who had access to the story topics search facility (yes vs no): were more positive about (mean scores 4.0 vs 3.6, *P* = .001) and more satisfied with the search facility (mean scores 7.3 vs 6.3, *P *< .001); were more positive about the number of search options (mean scores 2.3 vs 2.1, *P* = .04); were better enabled to find desired information (mean scores 3.3 vs 2.8, *P* = .001); were more likely to recommend the search facility to others or intend to use it themselves (mean scores 4.1 vs 3.5, *P* < .001); were more positive about how retrieved stories were displayed (mean scores 3.6 vs 3.2, *P* = .001); retrieved stories that better covered their information needs (mean scores 3.0 vs 2.6, *P* = .02); were more satisfied with the stories retrieved (mean scores 7.1 vs 6.4, *P* = .002); and were more likely to report an impact of the stories on coping with breast cancer (mean scores 3.2 vs 2.9, *P* =. 02). Three main effects were associated with use of the writer profile search (yes vs no): being more positive about (mean scores 3.9 vs 3.6, *P* = .005) and more satisfied with the search facility (mean scores 7.1 vs 6.5, *P* =. 01), and being more positive about how retrieved stories were displayed (mean scores 3.8 vs 2.9, *P* < .001). For satisfaction with the search facility, an interaction effect was found (*P* = .03): at least one of the two search facilities was needed for satisfaction.

**Conclusions:**

Having access to the story topics search facility clearly had the most positive effect on patient satisfaction and search success.

## Introduction

Patients value having access to stories of other patients as it provides them with emotional support, information, reassurance, and practical advice [1]. The Internet is a valuable resource for accessing stories because of its privacy and 24-hour availability without the need to leave one’s home [2]. Two well-known examples of web-based applications that include personal stories of patients are The Comprehensive Health Enhancement Support System (CHESS) [3] and The Database of Individual Patients’ Experiences of Illness (DIPEx) [4].

Studies of online patient stories have focused on several factors. These include why patients publish their stories online and what this means in a broader sociological context [5-7]. Wise et al [8] found that accessing personal stories in a computer-based patient support system had a positive effect on patients’ healthcare participation, which entailed participation preferences, confidence, and communication with their doctor. Little is known, however, about how patients search online for stories of other patients and whether they can find relevant ones.

Some qualitative studies have found that patients appreciate the ability to select stories of other patients of a particular age or who have opted for similar treatment [1,9]. In addition, searching by topics seems also to be of interest [10]. Some websites with patient stories provide a search facility to search for personal characteristics of the story writers and/or for topics written about in the stories [11-13]. However, to our knowledge, patients’ satisfaction and search success with these search facilities have not yet been studied.

In the present study, we examined which search facilities for patient stories resulted in satisfaction with the search process and the stories retrieved. We also studied the impact of the stories retrieved on coping with illness. Our expectation was that having a search facility would be an improvement compared with not having a search facility. Moreover, we expected that a combination of search facilities would result in higher satisfaction than a single search facility because a combination of search facilities may result in more opportunities to find a relevant story.

## Methods

### Design and Procedure

#### Study Design

We focused our study on patients with breast cancer. We contacted the board of The Amazones Foundation, which was founded by a group of young women with breast cancer to provide their peers with information and support. The Amazones Foundation developed a website for young women with breast cancer [14] that provides information and advice, a calendar of activities, an online support group, and links to other sites. The website also has a section with personal stories. Women can anonymously submit their own story to the site. The stories are presented alphabetically by writers’ nicknames. If a writer passes away, her story remains on the site accompanied by an obituary written by the website moderators.

**Figure 1:**
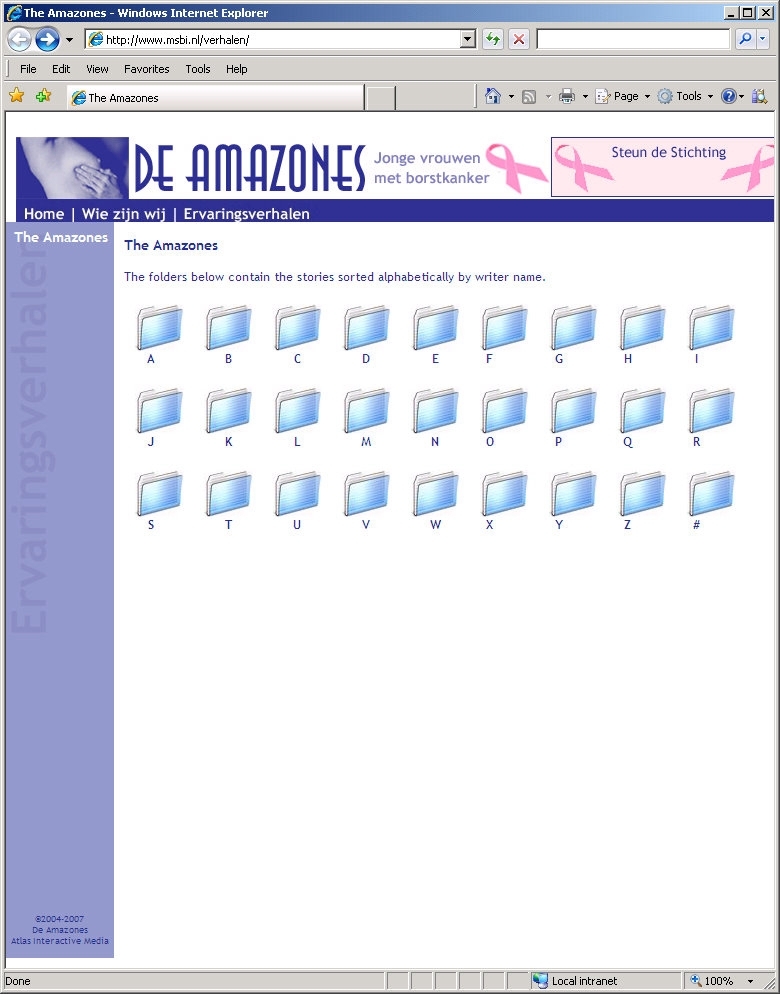
Screenshot of the control group search page

**Figure 2:**
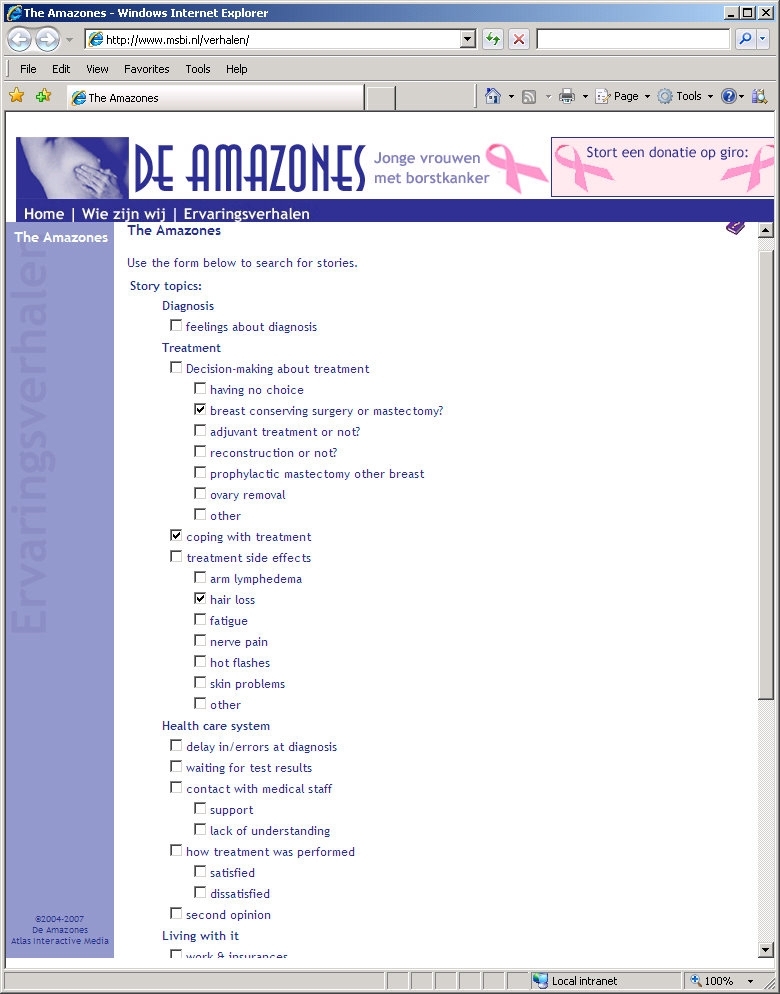
Screenshot of the story topics group search page

**Figure 3:**
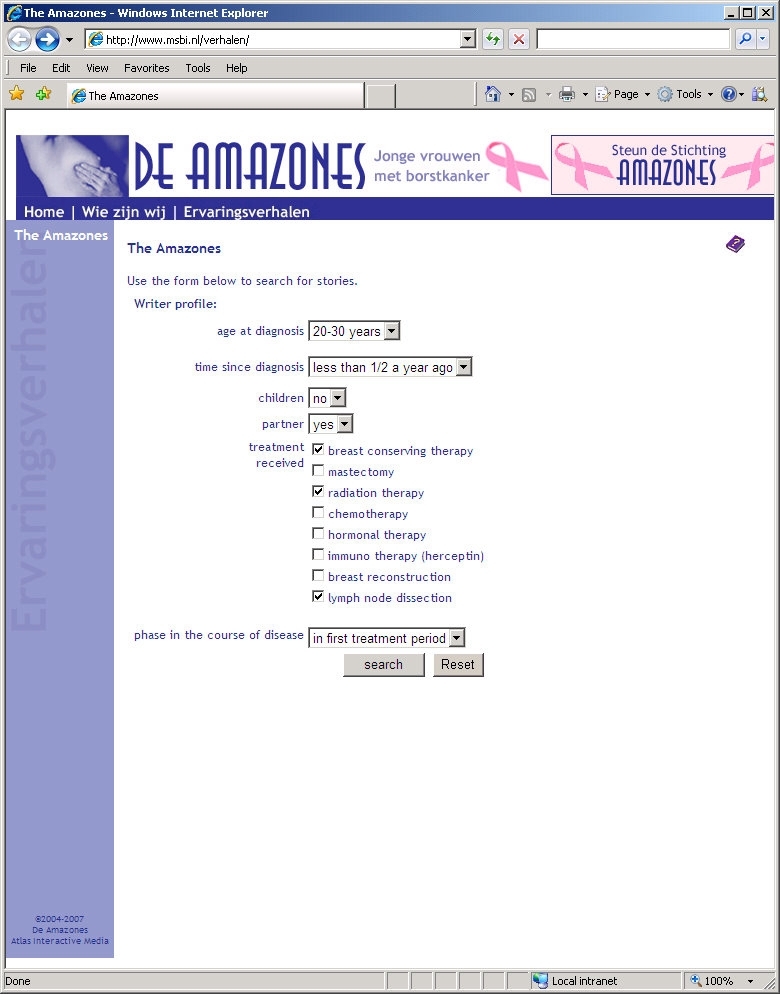
Screenshot of the writer profile group search page

**Figure 4:**
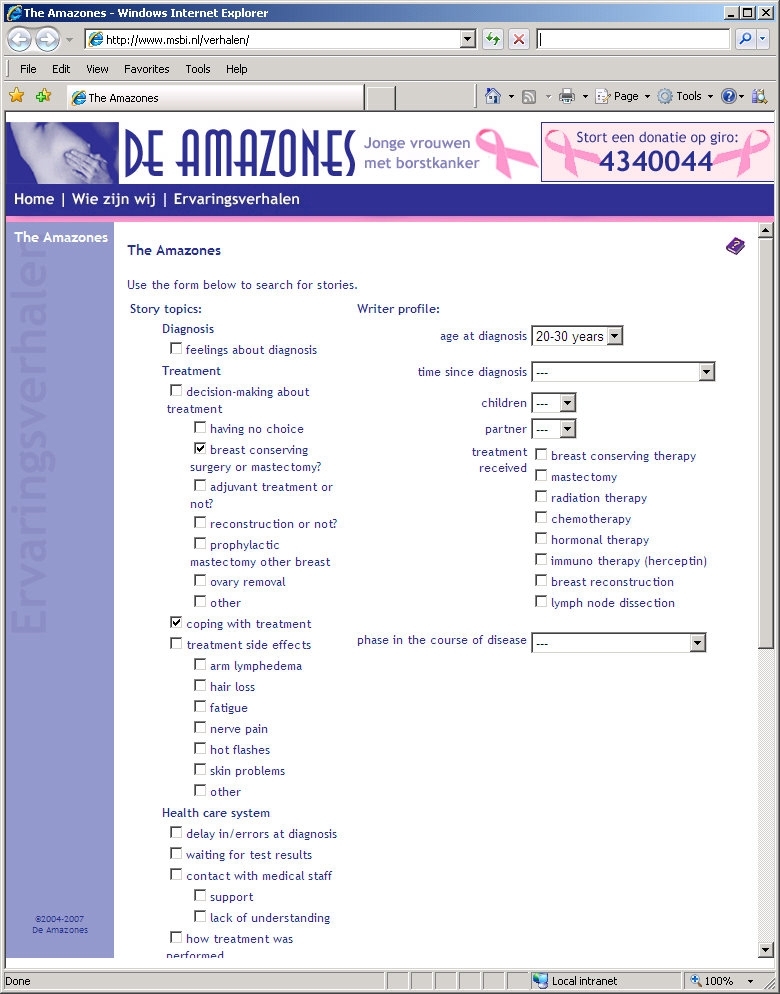
Screenshot of the combination group search page

We were granted permission by the board of The Amazones Foundation to conduct our study. In January 2007 we downloaded all 170 stories available at that time on their website for use in our study. We facilitated access to the stories in three ways: (1) with a search facility for story topics, (2) with a search facility for writer profiles, and (3) with a combination of these two search facilities. In addition, a control group could access the stories by means of the original alphabetical listing by story writer. We implemented these four ways of facilitating access to the stories on a separate study website. This resulted in four groups based on two independent factors: (1) no versus yes search by story topics, and (2) no versus yes search by writer profile (Table 1). In each of the four groups the same set of 170 stories could be searched. Figures 1 to 4 show screenshots of the search pages available to the four groups. We requested that the board of the Amazones Foundation not to participate in the study.

The present study is reported in accordance with the CHERRIES checklist, which is a checklist for reporting results of Internet e-surveys [15]. It was not registered as a clinical trial on ClinicalTrials.gov, a registry of clinical trials conducted around the world, because our study does not correspond to the definition of a clinical trial as provided in their glossary.

**Table 1:** The 2x2 factorial design of the study

		Writer Profile Search	
		No	Yes
Story Topics Search	No	Control group: see Figure 1	Writer profile group: see Figure 3
	Yes	Story topics group: see Figure 2	Combination group: see Figure 4

#### Recruitment Process

Recruitment announcements were disseminated online using banners on the websites of several Dutch patient and health organizations and offline using posters and flyers in waiting rooms of several hospitals. Dutch-speaking women with breast cancer were invited to participate irrespective of other personal characteristics. The offline recruitment announcements gave the URL of the study website, and the online announcements contained a hyperlink to the study website. The study was accessible to each visitor of the site, but only visitors who met the inclusion criteria were further directed to the informed consent page. After finishing the final questionnaire, participants could send a ready-made email message with the URL of the study website to other women who might be interested in participation. The study website was accessible in the period June through November 2007. During this time period, women could choose for themselves on what day and time they wanted to participate. No incentives were offered for participation.

#### Study Website

The first page of the study website provided the following information about the study: study objective, information about the researchers, study inclusion criteria, details about participation, expected time within which to complete the study, and contact details. When a website visitor chose to participate by clicking the button “I would like to participate”, two questions were presented to check whether the visitor met the inclusion criteria (ie, being female and having been diagnosed with breast cancer). If this was the case, the visitor was asked to read the informed consent statement. By agreeing with the informed consent statement, the visitor declared that her participation was voluntary, that she understood what participating entailed, and that she was aware of what data would be recorded. To agree with the informed consent statement, the visitor had to check the box “I agree” and then click on the button marked “Next”. After this the visitor was asked whether she was certain that she agreed with the informed consent statement. In this way, we assured that women did not automatically agree to participate. Before the participant was randomly assigned to one of the four groups, she was asked to provide a short description of the information she wanted to search for in the stories. Random assignment of each participant to a study group was carried out by an algorithm that was part of the study website. The chance of being assigned to a group was equal for all four groups, that is, 1 in 4 (except when more than one session was conducted from the same IP address; see the section below labeled “Technical Aspects”). Once assigned to a group, a participant could search for and read the stories as long as she liked. When finished with searching and reading she was asked to complete a final questionnaire posted on the study website about satisfaction with the search process and the stories retrieved and the stories’ impact on coping with breast cancer.

#### Ethical Aspects

Participants remained anonymous since no log-in, name, or address were required. In order to minimize traces on each client’s computer, no cookies were used. Recording of log data did not start until participants had agreed to the informed consent statement. Questionnaire responses were not saved until participants confirmed at the end of the final questionnaire that they agreed to submit their responses. Data were saved in a password protected SQL database only accessible to the researchers. Participants could stop at any time without receiving pop-ups or text when leaving the study website.

Before the start of the study, our research proposal (see Multimedia Appendix 1) was presented to the Ethical Committee of the Leiden University Medical Centre (archive number 06/43). The Committee concluded that our study involved no medical intervention and that we could proceed. Our intervention consisted of providing access to the stories already available on the website of the Amazones Foundation in several new ways.

### Development of Intervention Groups

#### Search Facilities

To develop the search facilities, all 170 stories were coded according to a coding scheme for story topics and a coding scheme for writer profile (Table 2). The topics and personal characteristics in the coding schemes were chosen because they had been used on other websites that contained breast cancer stories [11-13] or by other authors of studies in this field [1,9,10]. For the characteristic “phase in the course of disease,” the category “passed away” was assigned to stories that contained an obituary.

Participants could search for age using the categories: 20-30 years, 30-40 years, 40-50 years, and over 50 years. Participants could search for time since diagnosis using the categories: less than half a year ago, ½ - 1 year ago, 1-3 years ago, 3-5 years ago, and more than 5 years ago. To ensure that participants were aware of all search facilities, the search button was placed at the bottom of the page. In the groups with access to a single search facility, it was possible to search for more than one topic or more than one writer characteristic. In the combination group, participants could choose whether they wanted to search for story topics only, for writer characteristics only, or for both. Searching for more items simultaneously was based on the OR Boolean operator.

**Table 2:** Coding schemes for story topics and writer profile

Search Facility		Coding Scheme
**Story topics (domains)**		
	Diagnosis	Feelings about diagnosis
	Treatment	Decision-making about treatment: (1) having no choice, (2) breast conserving surgery or mastectomy?, (3) adjuvant treatment or not?, (4) reconstruction or not?, (5) prophylactic mastectomy of other breast, (6) ovary removal, (7) other
		Coping with treatment
		Treatment side effects:(1) arm lymphedema, (2) hair loss, (3) fatigue, (4) nerve pain, (5) hot flashes, (6) skin problems, (7) other
	Health care system	Delay in/errors at diagnosis
		Waiting for test results
		Contact with medical staff: (1) support, (2) lack of understanding
		How treatment was performed: (1) satisfied, (2) dissatisfied
		Second opinion
	Living with it	Work and insurances
		Family and friends: (1) support, (2) lack of understanding, (3) talking with and worrying about
		Body image and sexuality: (1) (partly) missing a breast, (2) partner’s reaction
		Pregnancy issues: (1) pregnant at diagnosis, (2) wanting to become pregnant after treatments
		Coping with breast cancer: (1) thinking (emotional-focused coping), (2) doing (problem-focused coping)
		Practical advices
		Concerns about heredity
		Coping with metastasized breast cancer
**Writer profile (personal characteristics)**		
	Age at diagnosis	Number of years
	Time since diagnosis	Number of months
	Partner	(1) No, (2) yes
	Children	(1) No, (2) yes
	Treatment received	(1) Breast conserving therapy, (2) mastectomy, (3) radiation therapy, (4) chemotherapy, (5) hormonal therapy, (6) immuno therapy (herceptin), (7) breast reconstruction, (8) lymph node dissection
	Phase in the course of disease	(1) In first treatment period, (2) free of cancer, (3) cancer for second time, (4) metastasized cancer, (5) passed away

#### Weight Assignment in Story Retrieval

For every search performed by the participants, a weight between 0 and 1 was assigned to each of the 170 stories in the database. If a story matched exactly with the search objectives, it received a weight of 1. Story weights were calculated with every new search. Therefore, the weight assigned to a story could change with every search.

In the story topics group, weights were calculated by dividing the number of topics found in a story by the number of topics that were searched for. For example, when a participant searched for four topics, all stories containing one of these four topics received a weight of ¼ (0.25).

In the writer profile group, a weight was assigned to each of the personal characteristics that a participant searched for. These weights were then multiplied with each other to calculate the weight of a story as a whole.

If the age of a writer fell in the age category that the participant was searching for, then “age” received a weight equal to 1. The more the age of the writer deviated from the age category that the participant was searching for, the lower the weight that “age” received. In a similar way, weights for “time since diagnosis” were assigned.

If the partner status of a writer exactly matched the partner status that the participant was searching for, then “partner” received a weight equal to 1. If the partner status of a writer is unknown, then this characteristic received a weight of 0.5 irrespective of the partner status that the participant was searching for. If the partner status of a writer was the opposite of the partner status that a participant was searching for, then this characteristic received a weight of 0.2. We did not assign a weight of 0 for the latter case because then the weight for the whole story would be 0. In similar ways, weights for the other categorical variables were assigned.

In the combination group, weight assignment was similar to that of the previous two groups or a multiplication of these two, depending on whether a participant searched for story topics only, for writer profile only, or for both.

#### Number of Stories Retrieved

It was decided to present participants with at least ten stories after each search. The total number of stories presented after a search depended on the distribution of the weights assigned to the stories. All stories with the same weight as the tenth story were presented because we saw no valid reason for presenting only a portion of the stories with that weight. For example, when five stories matched exactly with the search objectives, that is, weight equal to 1, 20 stories received a weight of 0.80, 45 stories received a weight of 0.60, and 100 stories received a weight of 0.40, then 25 stories (5 + 20) would be presented. Accordingly, if no stories exactly matched the search objectives, still at least ten stories were presented. The list of retrieved stories showed the extent to which the stories matched the search objectives. In this way we tried to present participants with neither too few nor too many stories.When participants did not fill in the search page, no stories were presented to them because we wanted to ensure that participants were aware of the search facility.

#### Story Display and Sequence

The retrieved stories were displayed as a list giving for each story the writer’s nickname and the story’s weight. Weights were represented as a number of pink ribbons. In addition, the search criteria that were fulfilled were given in each group, that is, the topics found, the writer’s characteristics, or both (see Multimedia Appendix 2). The list of stories was sorted by weight with the story with the heighest weight at the top. Stories with the same weight in the story topics group were displayed as follows. For each story, the percentage of text of the story relating to the topics the participant searched for was calculated. Stories with the highest percentage were ranked first. In the writer profile group, stories with the same weight were sorted by the age of the writers, and if age of writers was equal by time since diagnosis. Clicking on a story title from the list displayed the complete story.

### Final Questionnaire

#### Demographic and Disease Characteristics

Participants were asked to provide demographic information such as age, marital status, children, religion, education, and employment status. They were also asked to report characteristics of their cancer, such as time since diagnosis, type of diagnosis, metastases in axillary lymph nodes or other parts of the body, treatment undergone, and prognosis.

#### Use of the Internet and the Amazones Website (Before Study Participation)

The participants were asked to indicate their frequency of Internet use, the type of activities in which they engage on the Internet, and whether they had read stories of other patients on the Internet before. Moreover, they were asked to report how often they had visited the Amazones website before, how familiar they were with this website, and how many of the stories on this website they read before.

#### Satisfaction With the Search Process, the Stories Retrieved, and the Stories’ Impact on Coping With Breast Cancer

The constructs listed below were used to measure the three outcomes. Cronbach alphas were calculated using SPSS version 16.0 (SPSS Inc, Chicago, IL, USA) by conducting reliability analyses. Reverse phrased items were recoded. We found the internal consistency for each construct to be good or satisfactory (Cronbach alpha = 0.71 to 0.88). The items “overall satisfaction with the search facility” and “overall satisfaction with the stories retrieved” were answered using 10-point Likert scales; all other items were answered using 5-point Likert scales. For an overview of the items belonging to all the below mentioned constructs, see the Multimedia Appendix 3.

To measure satisfaction with the search process, 13 items were formulated (partially based on [16,17]). “Opinion about the search facility” was measured using 5 items (Cronbach alpha = .88). To measure “the extent to which the search options enabled finding information one was looking for,” 4 items were formulated (Cronbach alpha = .75). “Recommendation to others and future own use” was measured with 2 items (Cronbach alpha = .82). “Opinion about the number of search options” and “overall satisfaction with the search facility” were measured with 1 item each.

To measure satisfaction with the stories retrieved, 18 items were formulated (partially based on [16,17]). “Opinion about the stories retrieved” was measured with 6 items (Cronbach alpha = .71). To measure “opinion about the list of stories displayed after a search” 4 items were formulated (Cronbach alpha = .76). “The extent to which the stories retrieved covered one’s information need” was measured with 4 items (Cronbach alpha = .82) and “recommendation to others and future own reading” with 2 items (Cronbach alpha = .77). “Opinion about the number of stories retrieved” and “overall satisfaction with the stories retrieved” were measured with 1 item each.

“The stories’ impact on coping with breast cancer” was measured with 6 items (Cronbach alpha = .85) which were based on an extensive literature on coping [18-21]. Two of the items were formulated to measure problem-focused coping (“By reading the stories I have learnt things” and “By reading the stories I know what to do”), another two items were formulated to measure emotion-focused coping (“By reading the stories I am more able to understand my feelings” and “By reading the stories I can see that certain emotions are part of learning to live with breast cancer”), one item was formulated to measure reappraisal (“By reading the stories I view things in a different way”) and one item was formulated to measure social comparison (“By reading the stories I see that others have experienced the same things”). The existing validated coping scales, such as the Ways of Coping checklist [18,19] and the COPE inventory [21], could not be used because they were too general for our research question.

### Technical Aspects

We tested the usability and technical functionality of the study website, including the final questionnaire, multiple times, and we solved all appearing errors. During participants’ search processes, log data recorded how long participants surfed on the study website, how many searches they performed, how many stories they accessed, and how long the text of the stories was displayed on the screen. In the control group, clicking on a folder (A to Z) was regarded as performing a search, and subsequently clicking on a name was seen as accessing a story. Also, the time participants needed to fill in the final questionnaire was recorded.

Participants who were searching for or reading the stories were reminded to fill in the final questionnaire by a yellow figure on the left side of the screen with the text “Do not forget to complete the questionnaire,” which was highlighted every five minutes. Adaptive questioning was used to reduce the number and complexity of the questions. Questions were not randomized or alternated. The final questionnaire was distributed over five pages in the following sequence:(1) the search process, (2) the stories retrieved, including the stories’ impact on coping with breast cancer, (3) use of the Internet and the Amazones website, (4) disease characteristics, and (5) demographic characteristics. When participants clicked on the “Next” button at the end of a page, JavaScript was used to check for completeness. Unanswered questions were highlighted, and participants were asked to answer these. Yet, answering was not enforced, since by clicking on the “Next” button again, the next page was reached. Participants were not able to review and change their answers in previous parts to prevent a possible influence of questions asked later in the questionnaire.

Log data and questionnaire responses were saved automatically in an SQL database. In preparation for data analysis, sessions from the same IP address with a time interval of less than 20 minutes were merged, and those with a time interval of greater than 20 minutes were kept as two separate sessions. We assumed that in the former case the sessions were from the same participant and, in the latter, from different participants. Applying the first rule resulted in 23 merged sessions; the latter rule was applied to 6 pairs of sessions. Merging was possible because in all cases the questionnaire was filled out only once. Participants were only distinguished by IP address. A particular IP address was always assigned to the same intervention group. This was done to prevent women from participating multiple times when trying to get in another study group.

### Data Analysis

The data were imported into SPSS version 16.0. Differences in the log data between questionnaire completers and noncompleters were assessed using Mann-Whitney tests. The noncompleters were excluded from further analyses, since no questionnaire responses for this group were available. For the completers, there was no time frame for filling in the questionnaire. Differences between the four groups in baseline characteristics were assessed using Chi square tests, 1-way ANOVA, or Kruskal-Wallis tests (depending on variable type and skewness).

Kruskal-Wallis tests were performed to assess differences between the four groups in search behaviour (ie, the log data). Significant differences were examined further by performing post hoc tests. We chose to use Mann-Whitney tests with a Bonferroni correction, and as as the critical level of significance we used .05/6 = .008 because with four groups six comparisons were performed.

For each construct of the three outcome measures (satisfaction with the search process, the stories retrieved, and the stories’ impact on coping with breast cancer) a mean total score was calculated. A higher mean indicated a higher satisfaction or impact respectively. The effects of the search facilities on the constructs of the three outcome measures were examined using ANOVA with two independent factors (search facility for story topics yes/no; search facility for writer profiles yes/no) to assess possible main and interaction effects. This analytical approach was chosen in order to examine the effects of the two search facilities both independently and in combination. *P* values above .05 were considered not significant.

## Results

### Participant Statistics

**Figure 5:**
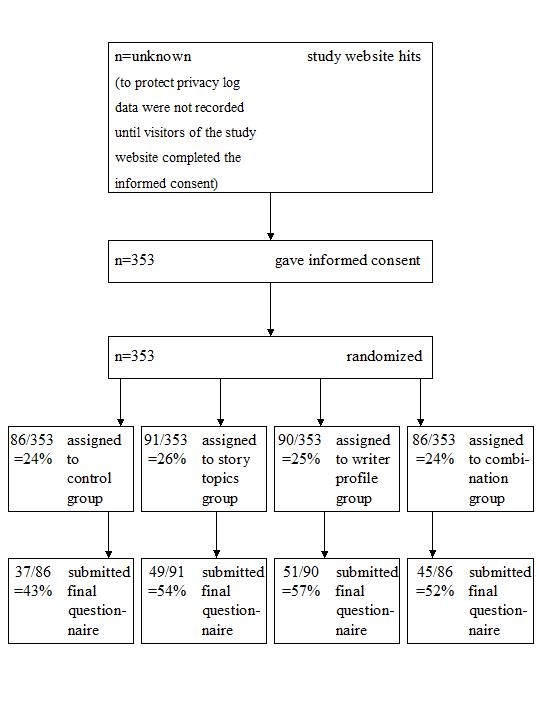
Flow of study participants

Informed consent was given by 353 people, of whom 182 (51.6%) completed the final questionnaire (Figure 5). No significant difference was found between the four groups in percentage questionnaire completers (χ^2^_3_= 3.7, *P* = .30). The mean time that participants needed to fill in the final questionnaire was 15.3 minutes (SD = 12.7; min = 5.0, max = 138.4). In comparison with questionnaire noncompleters, questionnaire completers spent less time visiting the study website (mean = 809.1 seconds vs 928.0 seconds, *P* < .001), but completers performed more searches (mean = 2.1 vs 1.7, *P* < .001), accessed more stories (mean = 6.6 vs 3.4, *P* < .001), and their mean reading time per story was longer (mean = 92.8 seconds vs 63.5 seconds, *P* < .001).

Table 3 shows the baseline characteristics of the questionnaire completers. No significant differences between the four groups were found for demographic and disease characteristics and use of the Amazones websites. With respect to use of the Internet, the writer profile group was less familiar with accessing fellow patients’ stories on the Internet.

**Table 3:** Baseline characteristics of the four groups

			Control Group (n = 37)^a^			Story Topics Group (n = 49)^a^		Writer Profile Group (n = 51)^a^			Combination Group (n = 45)^a^			*P* value^b^
			n		%	n	%	n		%	n		%	
**Demographic characteristics**														
	Age in years (mean, SD)		49.1 (7.5)			49.9 (8.3)		49.5 (9.4)			45.9 (9.9)			.12^c^
	Married or living together	Yes	29	78.4		38	77.6	40	78.4		34	75.6		.99
		No	8	21.6		11	22.4	11	21.6		11	24.4		
	Children	Yes	29	78.4		40	81.6	41	80.4		31	68.9		.45
		No	8	21.6		9	18.4	10	19.6		14	31.1		
	Religious	Yes	16	43.2		19	38.8	19	37.3		12	26.7		.44
		No	21	56.8		30	61.2	32	62.7		33	73.7		
	Higher professional education or university degree	Yes	11	29.7		18	36.7	23	45.1		15	33.3		.47
		No	26	70.3		31	63.3	28	54.9		30	66.7		
	Employed	Yes	22	59.5		24	49.0	31	60.8		26	57.8		.64
		No	15	40.5		25	51.0	20	39.2		19	42.2		

**Disease characteristics**														
	Time since diagnosis in months (mean, SD)		36.8 (45.8)			34.9 (41.4)		42.0 (41.8)			34.0 (37.3)			.49^d^
	Diagnosed with one tumour	Yes	23	62.2		35	72.9	37	72.5		32	71.1		.69
		No	14	37.8		13	27.1	14	27.5		13	28.9		
	Size of tumour	<2 cm	9	25.0		15	32.6	18	36.7		20	44.4		.32
		≥2 cm	27	75.0		31	67.4	31	63.3		25	55.6		
	Cancer in axillary lymph nodes at diagnosis	Yes	15	41.7		27	57.4	24	49.0		23	51.1		.56
		No	21	58.3		20	42.6	25	51.0		22	48.9		
	Metastases to other parts of the body	Yes	6	16.7		4	8.5	5	10.2		6	13.3		.68
		No	30	83.3		43	91.5	44	89.8		39	86.7		
	Breast conserving surgery	Yes	13	35.1		21	42.9	18	35.3		20	44.4		.71
		No	24	64.9		28	57.1	33	64.7		25	55.6		
	Mastectomy	Yes	19	51.4		24	49.0	34	66.7		30	66.7		.15
		No	18	48.6		25	51.0	17	33.3		15	33.3		
	Radiation therapy	Yes	22	59.5		28	57.1	25	49.0		20	44.4		.47
		No	15	40.5		21	42.9	26	51.0		25	55.6		
	Chemotherapy	Yes	24	64.9		35	71.4	31	60.8		26	57.8		.54
		No	13	35.1		14	28.6	20	39.2		19	42.2		
	Hormonal therapy	Yes	21	56.8		23	46.9	23	45.1		18	40.0		.50
		No	16	43.2		26	53.1	28	54.9		27	60.0		
	Cancer free	Yes	26	70.3		30	61.2	32	62.7		28	62.2		.83
		No	11	29.7		19	38.8	19	37.3		17	37.8		

**Use of the Internet and the Amazones website**														
	Daily Internet use	Yes	31	83.8		43	87.8	47	92.2		35	77.8		.23
		No	6	16.2		6	12.2	4	7.8		10	22.2		
	Familiar with searching online for specific information	Yes	36	97.3		46	93.9	48	94.1		43	95.6		.88
		No	1	2.7		3	6.1	3	5.9		2	4.4		
	Familiar with accessing fellow patients’ stories on the Internet	Yes	30	81.1		44	89.8	33	64.7		40	88.9		.005
		No	7	18.9		5	10.2	18	35.3		5	11.1		
	Visited the Amazones website at least once a month before participation	Yes	11	29.7		18	36.7	15	29.4		19	42.2		.52
		No	26	70.3		31	63.3	36	70.6		26	57.8		
	“Rather well” or “well” familiar with Amazones website	Yes	7	38.9		18	52.9	15	50.0		13	38.2		.56^e^
		No	11	61.1		16	47.1	15	50.0		21	61.8		
	Read half or more of the Amazones stories before	Yes	5	27.8		15	44.1	13	43.3		12	35.3		.62^e^
		No	13	72.2		19	55.9	17	56.7		22	64.7		

^a^N (%) is shown unless noted otherwise.

^b^ *P* values are for chi-square tests comparing the four groups unless noted otherwise.

^c^ *P* value for 1-way ANOVA test to compare the four groups with respect to age.

^d^ *P* value for Kruskal-Wallis test to compare the four groups with respect to time since diagnosis.

^e^Percentages and tests based on the number of participants who had previously visited the Amazones website: control group (n = 18), story topics group (n = 34), writer profile group (n = 30), combination group (n = 34).

### Search Behaviour

Table 4 shows that there were no differences between the four groups in time spent on the study website or in the number of searches performed. However, we found differences between the four groups in the number of stories accessed and in the mean reading time per participant per story. Post hoc tests (with a critical level of significance of *P* = .008 due to Bonferroni correction) showed that compared with the control group, fewer stories tended to be accessed in both the writer profile group (*P* = .01) and the combination group (*P* = .02). In addition, in the control group, the mean reading time per participant per story was shorter than in the writer profile group (*P* = .007) and tended to be shorter compared with the story topics group (*P* = .02) and the combination group (*P* = .009).

### Satisfaction With the Search Process

Table 5 shows that having access to the story topics search facility resulted in a more positive opinion about the search facility (1a), in a more positive opinion about the number of search options (1b), in being better enabled to find the information one was looking for (1c), in being more inclined to recommend it to others or to use it more often themselves in future (1d), and in a higher overall satisfaction with the search facility (1e), compared with not having access to this search facility (all comparisons were significant at *P* < .05). Having access to the writer profile search facility compared with not having access to this search facility resulted in a significantly more positive opinion about the search facility (1a), and in a significantly higher overall satisfaction score (1e).

An interaction effect was found for the overall satisfaction score (1e). When participants could search using the story topics, they were satisfied with this search facility regardless of whether (mean = 7.3, SD = 1.5) or not (mean = 7.2, SD = 1.4) they could also search with the writer profile. The effect of having access to the writer profile search facility when also having access to the story topics search facility was not significant (*P* = .90). However, when participants could not use the story topics to search the stories, they were more satisfied with having access to the writer profile as a search facility (mean = 6.8, SD = 1.6) compared with not having access to any search facility (mean = 5.7, SD = 2.3). The effect on satisfaction of having access to the writer profile search facility when not having access to the story topics search facility was significant (*P* = .009).

### Satisfaction With the Stories Retrieved

Having access to the story topics search facility resulted in a more positive opinion about the list of stories displayed after a search (2c), a greater extent to which the stories retrieved covered one’s information need (2d), and a higher overall satisfaction score with the stories retrieved (2f) compared with not having access to this search facility (Table 5). Having access to the writer profile search facility compared with not having access to this search facility resulted in a more positive opinion about the list of stories displayed after a search (2c).

There were no interaction effects observed in satisfaction with the stories retrieved.

### The Stories’ Impact on Coping With Breast Cancer

Table 5 shows that the stories retrieved using the story topics search facility had a greater impact on coping with breast cancer (3a). When analysing each of the six coping items individually, we observed that having access to the story topics search facility resulted in a significantly higher score for having learned things (3a.1).

**Table 4:** Comparison of the four groups for the search behaviour measures recorded by the log data

		Control Group (n = 37)	Story Topics Group (n = 49)	Writer Profile Group (n = 51)	Combination Group (n = 45)	*P* value^a^
Time spent on the study website in seconds	mean (SD)	754.00 (966.33)	984.55 (1278.94)	595.39 (630.04)	905.49 (1054.71)	.45
	median	496.00	634.00	389.00	636.00	
Number of searches	mean (SD)	3.89 (4.71)	1.88 (2.32)	1.53 (1.59)	1.69 (1.58)	.07
	median	2.50	1.23	1.24	1.28	
Number of stories accessed	mean (SD)	13.19 (18.98)	6.73 (6.36)	4.18 (4.53)	3.93 (3.61)	.01
	median	5.67	5.56	3.00	3.29	
Reading time per participant per story in seconds	mean (SD)	49.24 (54.65)	94.16 (94.90)	99.72 (128.27)	119.26 (113.00)	.02
	median	28.11	71.33	67.00	89.00	

^a^ *P* value for Kruskal-Wallis tests comparing the four groups with respect to the four search behaviour measures.

**Table 5:** Means (SD) of the constructs of the three outcome measures asked for in the final questionnaire by search factor

		Story Topics^a^			Writer Profile^a^			Interaction^a^
		Yes (n = 94)	No (n = 88)	*P* value^b^	Yes (n = 96)	No (n = 86)	*P* value^c^	*P* value^d^
**1. Satisfaction with the search process**								
	a. opinion about the search facility (range 1-5)	4.0 (0.7)	3.6 (1.1)	.001	3.9 (0.9)	3.6 (1.0)	.005	.21
	b. opinion about the number of search options (range 1-3)^e^	2.3 (0.6)	2.1 (0.8)	.04	2.3 (0.7)	2.2 (0.8)	.29	.23
	c. the extent to which search options enable finding information one was looking for (range 1-5)	3.3 (1.0)	2.8 (1.0)	.001	3.1 (1.0)	3.0 (1.0)	.27	.59
	d. recommendation to others and future own use (range 1-5)	4.1 (1.0)	3.5 (1.2)	< .001	3.9 (1.1)	3.8 (1.2)	.29	.13
	e. overall satisfaction with the search facility (range 1-10)	7.3 (1.4)	6.3 (2.0)	< .001	7.1 (1.6)	6.5 (2.0)	.01	.03
**2. Satisfaction with (the information in) the stories retrieved**								
	a. opinion about the stories retrieved (range 1-5)	3.5 (0.6)	3.4 (0.7)	.54	3.5 (0.6)	3.4 (0.7)	.36	.18
	b. opinion about the number of stories retrieved (range 1-3)^e^	2.3 (0.7)	2.1 (0.7)	.27	2.1 (0.7)	2.3 (0.7)	.18	.17
	c. opinion about the list of stories displayed after a search (range 1-5)	3.6 (0.9)	3.2 (1.2)	.001	3.8 (0.9)	2.9 (1.1)	< .001	.06
	d. the extent to which the stories retrieved covered one’s information need (range 1-5)	3.0 (1.0)	2.6 (1.0)	.02	2.7 (1.1)	2.9 (1.0)	.56	.91
	e. recommendation to others and future own reading (range 1-5)	4.1 (1.0)	3.8 (1.0)	.08	4.0 (1.0)	4.0 (1.0)	.67	.71
	f. overall satisfaction with the stories retrieved (range 1-10)	7.1 (1.5)	6.4 (1.7)	.002	6.7 (1.7)	6.9 (1.6)	.80	.35
**3. The stories’ impact on coping with breast cancer**								
	a. the stories’ impact on coping with breast cancer (range 1-5)	3.2 (0.9)	2.9 (1.0)	.02	3.0 (1.0)	3.1 (1.0)	.53	.71
	a.1. By reading the stories I have learned things (range 1-5)	3.0 (1.4)	2.5 (1.3)	.007	2.6 (1.4)	2.9 (1.3)	.24	.53
	a.2. By reading the stories I know what to do (range 1-5)	2.7 (1.2)	2.4 (1.2)	.14	2.5 (1.3)	2.6 (1.1)	.71	.70
	a.3. By reading the stories I am more able to understand my feelings (range 1-5)	3.0 (1.3)	2.6 (1.4)	.07	2.7 (1.4)	2.9 (1.3)	.70	.37
	a.4. By reading the stories I can see that certain emotions are part of learning to live with breast cancer (range 1-5)	3.8 (1.2)	3.5 (1.5)	.17	3.7 (1.4)	3.6 (1.3)	.82	.76
	a.5. By reading the stories I view things in a different way (range 1-5)	2.7 (1.3)	2.4 (1.3)	.16	2.4 (1.3)	2.7 (1.3)	.12	.16
	a.6. By reading the stories I see that others have experienced the same things (range 1-5)	4.1 (1.0)	3.9 (1.3)	.16	4.1 (1.1)	3.9 (1.1)	.28	.31

^a^ ANOVA with two independent factors (search facility for story topics yes/no; search facility for writer profiles yes/no). Higher means indicate better outcomes.

^b^ *P* value for possible main effect of story topics search

^c^ *P* value for possible main effect of writer profile search

^d^ *P* value for possible interaction effect between story topics search and writer profile search

^e^ Asked on a 5-point scale but for analysis recoded into 3-points (see also Multimedia Appendix 3)

## Discussion

### Principal Findings

To our knowledge, this study is the first randomized controlled experiment with a 2x2 factorial design that examined search facilities for accessing online patient stories. We observed that the story topics search factor had a strong impact on patient satisfaction and search success: participants were the most satisfied with this search facility and the stories retrieved. Also, the stories retrieved had a greater impact on coping with breast cancer. The effect of the writer profile search factor was limited. This search facility resulted only in a few effects, predominantly on satisfaction with the search process. The two search factors combined generally had no amplified effect on patient satisfaction or search success as we only had one significant interaction.

These findings are contrary to our expectation, which was that the combined search facilities (the interaction) would outperform a single search facility because this combination is more complete and differentiated resulting in greater opportunities to find a relevant story. Apparently, this quantity argument seems to be less important than the type of the search facility (quality). In line with our expectation was that a single search facility was an improvement compared with the alphabetically listed stories in the control group.

Participants in the three search facility groups accessed fewer stories and read longer per accessed story compared with the control group. An explanation for this might be that the stories retrieved in the search facility groups were more relevant to the participants. A search facility probably increases the proportion of the documents retrieved relevant to the user's information need [22].

The story topics search facility resulted not only in participants being more satisfied with the search process, but also in participants retrieving stories that better covered their information needs and retrieving stories from which they learned more. Patients might use online stories predominantly for information, and, therefore, the topics described in the stories might be more important for them than the writer’s profile. Patients’ profiles might be more important when seeking face-to-face contact. This difference between seeking information and seeking contact has also been noted by Bennenbroek et al [23] in their research on social comparison.

Our observation that the writer profile search facility compared with not having this search facility resulted in a more positive opinion about the search facility and in a higher overall satisfaction with the search facility is in line with the results of Rozmovits and Ziebland [1]. They found in interviews that patients positively evaluated the ability to select other patients of a particular age, stage of illness, or patients who were long-term survivors or who had opted for similar treatment. However, although our study also showed that participants were more satisfied with this search facility, they were not more satisfied with the stories retrieved using this facility.

### Limitations of the Present Study

A considerable number of participants performed searches but did not complete the questionnaire. Compared with completers, noncompleters spent more time on the study website while they performed fewer searches, accessed fewer stories, and spent less time reading per story. Noncompleters might not have been sure about how to use the search facilities, or they might not have been as interested. Yet, we could not empirically evaluate these hypotheses nor perform any statistical analyses, since we had no further information about noncompleters. The number of completers and noncompleters was evenly distributed over the four study groups. Therefore, we believe that potential bias equally affected all four groups. In addition, the direction of the bias is probably twofold: dissatisfied participants might have stopped or they might have completed the questionnaire to express their annoyance.

More than half of the participants who completed the questionnaire (63.7%) had previously visited the Amazones website. This could introduce bias because participants familiar with the original disclosure of stories might be especially satisfied with the new search facilities. However, frequency of visiting the Amazones site, knowing the site “rather well” or “well,” and the number of Amazones stories read before, were all evenly distributed over the four study groups. Therefore, we do not think this previous experience with the Amazones website affected the results.

Since the experiment was conducted completely online [24], we cannot verify that all participants indeed had (or had had) breast cancer. However, we targeted this group for recruitment and asked relevant questions before randomization. We assume that all participants were sincere, because overall they spent 15 minutes filling in the final questionnaire. This suggests that participants were interested in the subject matter.

A limitation of the design was a possible confounding between type of search facility (story topics, writer profile) and number of search options (17 topics, 6 personal characteristics). The story topics search might have been more appreciated because it was more extensive than the writer profile search. However, an argument against this reasoning is that the most extensive search facility (ie, story topics in combination with writer profile) was not the most favourite.

In addition, one could question the content of the search facilities. Were the most appropriate topics and personal characteristics included in the facilities? Yet, the topics and characteristics we used were chosen based on other websites containing breast cancer stories [11-13] and other studies in this field [1,9,10].

A final limitation is that participants may have been annoyed when stories were presented that did not exactly match their search objectives. However, a search resulting in no stories could also be a cause of annoyance. This is why we chose to present at least ten stories after each search. In order to ease interpretation of the resulting list of stories, weights (as pictured in the form of pink ribbons) were used to indicate to what extent a story matched with the search objective.

### Conclusions and Practical Implications

Earlier studies have shown that patients can benefit from stories of other patients, and that the Internet is an important source of these stories. Our current study suggests that a story topics search facility would be most helpful to patients. With a story topics search facility, participants were better enabled to find the information they were looking for. Also, they retrieved stories that more closely covered their information needs and they learned more from the stories retrieved.

Thus, patient organisations or website developers that offer patient stories on their websites can best provide access to them using a story topics search facility. However, constructing such a search facility is very time consuming and labour intensive since stories have to be coded for content. An efficient method might be to use a system analogous to social bookmarking/tagging [25] in which story readers assign keywords or tags to the stories, and the keywords or tags that are most often assigned are seen as most important in describing the content. Another possibility is to construct a list of items from which writers can compose descriptions of their stories. Finally, stories could also be classified by automatic full text indexing or clustering. This will be the subject of our next study.

## Multimedia Appendix 1

Research proposal (in Dutch) as presented to the ethical committee before the start of the study

## Multimedia Appendix 2

Screenshots illustrating the search page and the list of retrieved stories for each of the 4 search conditions (ie, control, story topics, writer profile, and combination condition)

## Multimedia Appendix 3

An overview of the constructs and items belonging to the three main outcome measures
