# Supplementary material for: How Breast Cancer Patients Want to Search for and Retrieve Information From Stories of Other Patients on the Internet: an Online Randomized Controlled Experiment
Source: J Med Internet Res. 2010 Mar 9;12(1):e7. doi: 10.2196/jmir.1215 (PMC2855205; doi:10.2196/jmir.1215)
Supplement: Supplementary file 1 [file jmir_v12i1e7_app1.pdf]

Multimedia Appendix I:
Research proposal (in Dutch) as presented to the Ethical Committee before the start of the study.

Appendix to:
Overberg R, Otten W, De Man A, Toussaint P, Westenbrink J, Zwetsloot-Schonk B. How breast cancer patients want to search for and retrieve information from stories of other patients on the Internet: an online randomised controlled experiment.

Effect van het op diverse manieren toegankelijk maken van online lotgenootverhalen op tevredenheid met het zoekproces en op bruikbaarheid van de gevonden verhalen: een gerandomiseerd, gecontroleerd experiment bij borstkankerpatiëntes

Onderzoeksvoorstel (concept)

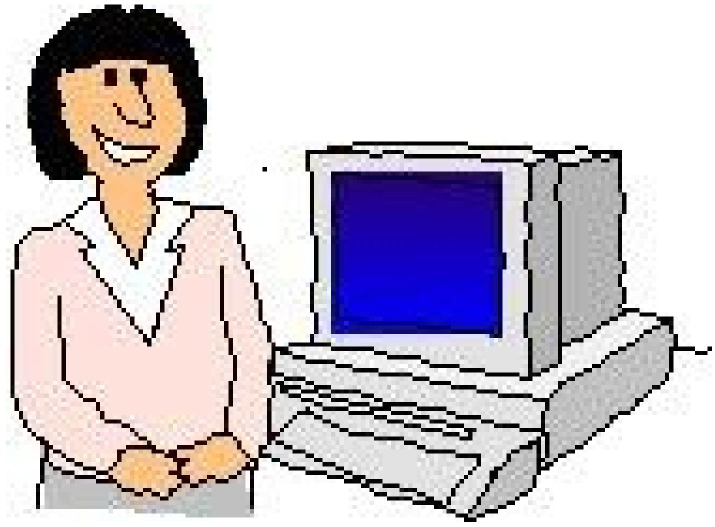

Auteur: Regina Overberg
Datum: juli 2006

Inhoudsopgave

Onderzoeksvoorstel (concept).. 2

.... 2

Inhoudsopgave... 3

Betrokkenen.. 5

Probleemstelling.. 6

- Doelstelling.. 7
- Vraagstelling.. 8

Relevantie... 8

- Theoretische/wetenschappelijke relevantie... 8
- Maatschappelijke relevantie.. 8

Object en begrenzing.. 8

Design.. 8

- Omschrijving.. 8
- Waarom dit design?... 9
- Waarom geen andere designs?.. 9
- 10

Deelneemsters... 11

- Inclusiecriteria... 11
- Werving... 12

Onderzoeksgroepen.. 13

- Wat wordt van de deelneemsters gevraagd?... 13
- Controlegroep... 14
- Interventiegroepen.. 14
- Hoe wordt het zoeksysteem voor de interventiegroepen gerealiseerd?........... 16
- Randomisatie en inlogprocedures... 18

Metingen.. 19

- Waar vinden de metingen plaats?.. 19
- Voormeting.. 19
- Meting tijdens interventie.. 20
- Nameting... 20

Techniek.. 21

Analyse en rapportage.. 22

Planning.. 22

Expertise, voorgaande activiteiten en producten..23

Eigen publicaties (gerelateerd aan het Narrator-project, op alfabet).......................24

Betrokkenen

Naam	*Organisatie*	*Functie*	*Rol*
Diana Gosens<br>Judith Westenbrink	Stichting Amazones[1]	Bestuur stichting; contactpersonen	Adviseur
Pieter Toussaint	LUMC,<br>Klinische Informatiekunde[2]	Senior onderzoeker	Projectleider Narrator
Bertie Zwetsloot-Schonk	LUMC,<br>Klinische Informatiekunde	Hoofd/ Hoogleraar	Eindverantwoordelijke
John Verhoef	LUMC,<br>Klinische Informatiekunde en Fysiotherapie	Onderzoeker	Adviseur
Wilma Otten	LUMC,<br>Medische Besliskunde[3]	Psycholoog/ onderzoeker	Adviseur
Andries de Man	LUMC,<br>Klinische Informatiekunde	COO-ontwikkelaar[4]	Technische ondersteuning
Regina Overberg	LUMC,<br>Klinische Informatiekunde	Promovendus	Onderzoeker Narrator

[1] Stichting Amazones, opgericht in januari 2005, voor en door jonge vrouwen met borstkanker. Zie ook: http://www.de-amazones.nl
[2] Leids Universitair Medisch Centrum, Klinische Informatiekunde. Zie ook: http://www.lumc.nl/3020
[3] Leids Universitair Medisch Centrum, Medische Besliskunde. Zie ook: http://www.lumc.nl/2050
[4] COO= Computer Ondersteund Onderwijs

Probleemstelling

De diagnose kanker heeft een enorme impact op de patiënt, die te maken krijgt met allerlei fysieke, psychologische en sociale gevolgen. Na behandeling en (soms helaas slechts tijdelijke) genezing houden de problemen vaak aan. Veel patiënten hebben te kampen met extreme vermoeidheid, pijn, angst dat het terugkomt, onzekerheid over de toekomst, moeizame relaties met familie en vrienden, en problemen met het weer aan het werk gaan (1). Ook hebben patiënten vaak het gevoel de controle te verliezen en/of het gevoel bedreigd te worden in hun eigenwaarde (2). Bovendien verandert kanker vaak de sociale rol en identiteit van een persoon, doordat de zieke persoon andere activiteiten heeft en andere gedachten ontwikkelt en doordat hij/zij anders wordt bekeken door mensen in zijn/haar omgeving (3).

De hierboven beschreven impact van kanker ontwricht het levensverhaal van een persoon. Elke persoon construeert – meestal onbewust – zijn/haar levensverhaal door alle gebeurtenissen en ervaringen aan elkaar te koppelen tot een samenhangend en kloppend geheel. De diagnose kanker verstoort dit samenhangende en kloppende geheel, met het gevolg dat de kankerpatiënt zijn/haar levensverhaal moet repareren door het construeren van een *illness story* (4;5). Met behulp van hun *illness story* kunnen patiënten: a) zin geven aan gezondheid en ziekte, b) controle behouden tijdens fysieke en psychologische verliezen, c) zich een nieuwe identiteit en sociale rol aanmeten die ze hebben gekregen als gevolg van de ziekte, en d) beslissingen over hun gezondheid maken (6). Verschillende onderzoeken hebben aangetoond dat het schrijven over ingrijpende gebeurtenissen -zoals ziek worden/zijn- positieve effecten heeft op de schrijver (7;8). Wij denken dat een *illness story* niet alleen een positief effect heeft op de schrijver van het verhaal, de kankerpatiënt, maar ook op de lezer die in dezelfde situatie verkeert als de schrijver, een lotgenoot. Lotgenoten die moeite hebben met het repareren van hun levensverhaal, kunnen *illness stories* van andere patiënten gebruiken als een voorbeeld van hoe het levensverhaal kan worden gerepareerd.

Verder bieden *illness stories* informatie, herkenning, en manieren om de ziekte te accepteren en zich eraan aan te passen (9). Online lotgenotencontact -waarbij *illness stories* worden uitgewisseld- verminderde bij borstkankerpatiënten depressie (10;11), stress (10), en reactie op pijn (11). Bovendien werd online lotgenotencontact nuttig gevonden (12), en hielp het patiënten beter om te gaan met de ziekte (13).

Vandaag de dag kan een groot aantal *illness stories* gevonden worden op het internet. Een voordeel van internet is dat *illness stories* gelezen kunnen worden op een tijd en een plaats die de lezer het beste uitkomen, en dat de lezer anoniem kan blijven (14). Echter, nadelen van internet zijn dat het vaak onduidelijk is voor de lezer of *illness stories* geschreven zijn door echte patiënten (15), en of de medische informatie in de *illness stories* betrouwbaar is (14). Uit ons onderzoek naar hoe Nederlandstalige *illness stories* van borstkankerpatiëntes worden aangeboden op internet komt naar voren dat er noch zoekmogelijkheden zijn naar de inhoud van verhalen met behulp van trefwoorden noch naar persoonskenmerken van schrijfsters van verhalen (16). Wij denken dat dit gebrek aan zoekmogelijkheden het vinden van *illness stories* over relevante onderwerpen geschreven door vergelijkbare lotgenoten bemoeilijkt, terwijl *illness stories* die aan deze twee criteria voldoen juist het meest nuttig zullen zijn bij het repareren van het eigen levensverhaal.

Om te achterhalen wat de wensen en behoeften van borstkankerpatiëntes zijn met betrekking tot *illness stories* (lotgenootverhalen) op het internet, hebben we interviews afgenomen (17). Uit deze interviews komt naar voren dat borstkankerpatiëntes in lotgenootverhalen vooral willen lezen over hoe lotgenoten zijn omgegaan met emoties, hoe ze zijn omgegaan met de invloed van kanker op het dagelijks leven, en hoe ze zijn omgegaan met lichamelijke ongemakken. De meeste geïnterviewden willen de lotgenootverhalen gepresenteerd krijgen als verhaalfragmenten over specifieke onderwerpen bestaande uit alleen tekst. Sommige geïnterviewden willen daarbij door kunnen klikken naar het complete verhaal waaruit het fragment afkomstig is. Ook willen sommige geïnterviewden graag dat de tekst ondersteund wordt door geluid- en video-opnamen van de patiënte die haar verhaal vertelt. De meerderheid van de geïnterviewden wil verhalen kunnen selecteren op basis van een aantal persoonskenmerken van schrijfsters, te weten: ondergane behandeling, leeftijd, het al dan niet hebben van uitzaaiingen, tijd verstreken sinds diagnose, en het al dan niet hebben van kinderen (17).

De Stichting Amazones heeft op 11 april 2006 126 verhalen op hun site staan, die gevonden kunnen worden door in het menu *Ervaringsverhalen* te kiezen. Op de pagina *Ervaringsverhalen* kan vervolgens gekozen worden voor: *De Amazones*, *De familie*, *De partners*, en *De vrienden*. Onder het kopje *De Amazones* zijn verhalen te vinden die geschreven zijn door borstkankerpatiëntes zelf; dit zijn er 123. Onder de andere drie kopjes zijn verhalen te vinden die geschreven zijn door respectievelijk familieleden (0 verhalen), partners (2 verhalen), en vrienden (1 verhaal) van borstkankerpatiëntes. Onder elk van de vier kopjes zijn de voornamen/nicknames van de schrijvers/schrijfsters alfabetisch gerangschikt. Door op een naam te klikken wordt het volledige verhaal getoond. Elders op de website (onder *Wie zijn wij* en vervolgens *De Amazones*) kunnen profielen van de schrijvers/schrijfsters opgezocht worden, waarin onder andere zijn opgenomen: geboortejaar, relatie of burgerlijke staat, kinderen, maand-jaar van diagnose, en ondergane behandelingen. De verhalen en de profielen op de website van Stichting Amazones zijn momenteel niet doorzoekbaar met behulp van trefwoorden. Wel kan een bezoeker via de zoekfunctie (op trefwoord) van het forum, dat ook aangeboden wordt op de site, relevante postings vinden van lotgenoten. Vervolgens kan de bezoeker het profiel en het ervaringsverhaal van de betreffende lotgenoot opzoeken om daar verder te lezen (18).

Uit de bovenstaande uiteenzetting komt naar voren dat de manier waarop lotgenootverhalen momenteel op internet worden aangeboden (18) niet overeenstemt met de wensen en behoeften van borstkankerpatiënten op dit gebied (17).

Doelstelling

Het onderzoeken van het effect van het op vier verschillende manieren aanbieden van online lotgenootverhalen aan borstkankerpatiëntes op tevredenheid met het proces van zoeken en op bruikbaarheid van de gevonden verhalen.

De vier manieren van aanbieden zijn:
A. op alfabet van auteursnaam;
B. met een zoekformulier naar inhoud;
C. met een zoekformulier naar persoonskenmerken van schrijfsters; en,
D. met zowel een zoekformulier naar inhoud als naar persoonskenmerken van schrijfsters.

Vraagstelling

Voor elke groep worden drie vragen beantwoord:

1. Hoe zoeken borstkankerpatiëntes naar verhalen (zoekgedrag)?;
2. Hoe tevreden zijn borstkankerpatiëntes met het proces van zoeken?; en,
3. Hoe bruikbaar zijn de gevonden verhalen voor de borstkankerpatiëntes (zoals perceived control, coping met de ziekte, repareren eigen illness story)?

Relevantie

Theoretische/wetenschappelijke relevantie

Dit onderzoek levert kennis op over het zoekgedrag en de zoekwensen van borstkankerpatiëntes met betrekking tot lotgenootverhalen op internet, en over wat lotgenootverhalen borstkankerpatiëntes te bieden hebben tijdens (en na) hun ziek zijn. Deze kennis zou gebruikt kunnen worden bij theorievorming op het gebied van mens-machine interactie en/of op het gebied van coping met een levensbedreigende ziekte.

Maatschappelijke relevantie

Door de gevonden kennis in de praktijk toe te passen (dat wil zeggen door de lotgenootverhalen zodanig te ontsluiten dat het beter aansluit bij wensen en behoeften van borstkankerpatiënten en door de set verhalen zodanig samen te stellen dat patiënten er al hun behoeftes uit kunnen halen) zullen patiënten meer baat hebben bij de lotgenootverhalen.

Object en begrenzing

Het op verschillende manieren ontsluiten van de *Ervaringsverhalen* van *De Amazones* op de website van de Stichting Amazones (http://www.de-amazones.nl) vormt het object van dit onderzoek. Het onderzoek wordt uitgevoerd bij vrouwen die borstkanker hebben of hebben gehad. Uitkomstmaten van het onderzoek zijn: tevredenheid met het zoekproces en bruikbaarheid van de gevonden verhalen.

Er wordt *geen* narrative analysis gedaan. Dat wil zeggen dat er *niet* onderzocht wordt wat voor bedoeling elke schrijfster met haar verhaal had, waarom een schrijfster heeft gekozen voor een bepaalde opbouw, enzovoorts. Verder wordt er ook *geen* algemeen onderzoek gedaan naar de website van Stichting Amazones, waarin vragen beantwoord worden als: hoeveel bezoekers heeft de website, welke delen van de website worden het meest aangeklikt, enzovoorts.

Design

Omschrijving

Er wordt gekozen voor een gerandomiseerd experiment met drie interventiegroepen en een controlegroep (zie figuur 1). Er worden twee factoren onderzocht (wel/geen zoekmogelijkheid naar inhoud en wel/geen zoekmogelijkheid naar persoonskenmerken

schrijfsters), dus eigenlijk komt het design neer op een *2x2 factorial design*. De deelneemsters worden geworven uit de populatie vrouwen die borstkanker hebben of hebben gehad en die geïnteresseerd zijn in lotgenootverhalen. In elke groep wordt aan deelneemsters gevraagd om in de ervaringsverhalen te zoeken naar hun eigen informatiebehoefte. Voor en na de interventie wordt aan deelneemsters gevraagd een vragenlijst in te vullen. Tijdens de interventie worden de acties van de deelneemsters vastgelegd door middel van log-files. De nameting wordt vergeleken tussen de vier groepen, zodat gekeken kan worden wat voor effect het heeft als ervaringsverhalen op verschillende manieren aangeboden worden.

Waarom dit design?

Waarom randomiseren? Dit zorgt ervoor dat de vier groepen in het experiment vergelijkbaar zijn. Door het randomiseren - toewijzing aan één van de groepen op basis van toeval - is de kans op systematische verschillen tussen de groepen gering. Wel kunnen er toevallige verschillen tussen de groepen ontstaan, maar daar kan in de analyse voor gecorrigeerd worden. Matchen is ook een manier om vergelijkbare groepen te krijgen, maar kent enkele nadelen: het is bewerkelijk; vooraf moeten de gegevens die nodig zijn om te kunnen matchen verzameld worden (lange vragenlijsten voor de deelneemsters); en er zijn verborgen variabelen (variabelen waarvan je je als onderzoeker niet bewust bent, maar die je toch gelijkmatig over de groepen verdeeld wilt hebben). Daarom wordt gekozen voor randomiseren.

Waarom een controlegroep? Dit zorgt ervoor dat je als onderzoeker de score op de nameting kent voor de situatie waarin je niks hebt gemanipuleerd. Als de scores op de nameting in de interventiegroepen verschillen van die in de controlegroep mag dat toegeschreven worden aan de interventie. Zonder controlegroep weet je niet of een gevonden effect komt door de interventie of door andere voor jou onbekende, externe factoren.

Waarom drie interventiegroepen? We zijn geïnteresseerd in twee factoren: het wel/niet hebben van een zoekmogelijkheid naar inhoud en het wel/niet hebben van een zoekmogelijkheid naar persoonskenmerken van schrijfsters. Door drie interventiegroepen te maken - twee groepen met elk van de factoren apart en één groep met beide factoren gecombineerd - kunnen we uitspraken doen over het effect van elk van de twee zoekmogelijkheden apart en over het effect van de combinatie van zoekmogelijkheden.

Waarom geen andere designs?

Waarom de interventiegroepen niet eerst de verhalen op alfabet laten zien en laten beoordelen, en daarna de interventie aanbieden (zgn. *within-subjects design*)? Voordelen hiervan lijken te zijn dat deelneemsters een referentie hebben bij het beoordelen van de interventie (ze kunnen de verhalen met zoekmogelijkheden vergelijken met de eerder geziene verhalen op alfabet) én dat elke deelneemster haar eigen controle is. Toch zijn er een drietal belangrijke redenen om dit niet te doen.

Ten eerste dienen deelneemsters dan twee keer in dezelfde set verhalen hun informatiebehoefte op te zoeken. Stel dat ze in de verhalen op alfabet reeds gevonden hebben waarnaar ze op zoek waren, dan is het erg onnatuurlijk om in dezelfde set verhalen (alleen anders toegankelijk gemaakt) nog eens te zoeken naar iets dat ze al gevonden

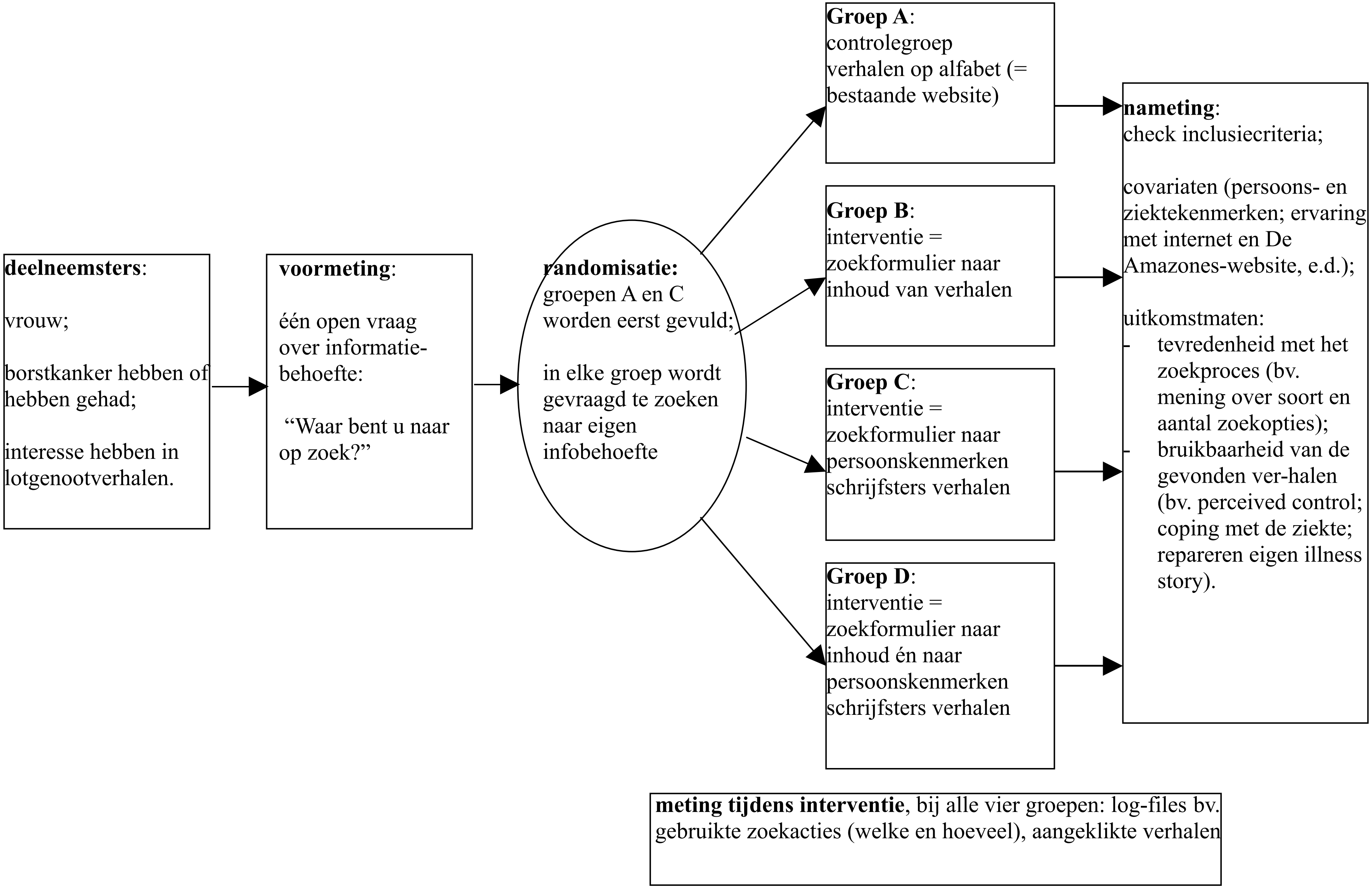

Figuur 1. Onderzoeksdesign: een gerandomiseerd experiment met drie interventiegroepen en een controlegroep.

hebben. Om deze onnatuurlijke situatie te voorkomen, zouden ze in de verhalen met zoekmogelijkheden naar andere informatie kunnen gaan zoeken dan dat ze gedaan hebben in de verhalen op alfabet. Echter, het voordeel van dit design - dat elke deelneemster haar eigen controle is - gaat dan niet meer geheel op. Ook als de deelneemsters in de verhalen op alfabet niet gevonden hebben waarnaar ze op zoek waren, zijn ze er toch door beïnvloed op het moment dat ze dezelfde set verhalen (alleen anders toegankelijk gemaakt) voor zich krijgen.

Ten tweede is het onwaarschijnlijk dat de deelneemsters louter de verhalen op alfabet gebruiken als referentie. De deelneemsters hebben vast al vele malen op internet gesurft en al diverse websites bezocht waarop lotgenootverhalen staan. Zij hebben dus al een mening over lotgenootverhalen op websites. De deelneemsters zullen dus de verhalen op alfabet niet als referentie gebruiken, terwijl je dat als onderzoeker wel aanneemt. Dit kan leiden tot verkeerde interpretaties.

Ten derde zijn deelneemsters beperkter in het beoordelen van de verhalen met zoekmogelijkheden (interventie) als ze eerst de verhalen op alfabet hebben moeten beoordelen. Stel ze geven de verhalen op alfabet het rapportcijfer 7, en de verhalen met zoekmogelijkheden willen ze een hoger rapportcijfer geven, dan kunnen ze alleen nog maar de rapportcijfers 8-10 geven. Als ze niet eerst de verhalen op alfabet hebben beoordeeld dan kunnen ze de verhalen met zoekmogelijkheden de rapportcijfers 1-10 geven. Ze hebben dus meer ruimte hun oordeel te geven als ze niet eerst de verhalen op alfabet beoordeeld hebben.

Waarom niet de helft van de deelneemsters eerst de verhalen op alfabet laten beoordelen en vervolgens de verhalen met zoekmogelijkheden, en de andere helft van de deelneemsters eerst de verhalen met zoekmogelijkheden laten beoordelen en vervolgens de verhalen op alfabet (zgn. *cross-over design*)? Voordelen hiervan lijken te zijn dat het makkelijker is voor deelneemsters omdat ze twee situaties met elkaar kunnen vergelijken én dat elke deelneemster haar eigen controle is. Er zijn vier redenen om dit niet te doen. De eerste drie redenen zijn gelijk aan de hierboven genoemde om geen *within-subjects design* te gebruiken. De vierde reden is dat je (te) veel groepen krijgt, omdat we zowel het effect van de zoekmogelijkheid naar inhoud, als het effect van de zoekmogelijkheid naar persoonskenmerken van schrijfsters, als de combinatie van beide willen onderzoeken.

Deelneemsters

Inclusiecriteria

Als inclusiecriteria worden gehanteerd: borstkanker hebben of hebben gehad; vrouw zijn; geïnteresseerd zijn in lotgenootverhalen. Waarom deze inclusiecriteria?

- Borstkanker hebben of hebben gehad.

Dit onderzoek is onderdeel van een groter onderzoeksproject, Narrator, waarin de case borstkanker wordt onderzocht. Borstkankerpatiënten zijn dus de doelgroep.

- Vrouw zijn.

Ook mannen kunnen borstkanker krijgen en hebben wellicht ook behoefte aan lotgenootverhalen via internet. Aan dit onderzoek doen alleen vrouwen mee en dat heeft twee redenen. Ten eerste zijn interviews in het kader van Narrator - waarvan de resultaten geleid hebben tot de interventiegroepen - ook alleen bij vrouwelijke borstkankerpatiënten afgenomen. Ten tweede richt de website van De Amazones zich alleen op vrouwen; er staan geen verhalen van mannelijke borstkankerpatiënten op.

- Geïnteresseerd zijn in lotgenootverhalen.

We beogen in het Narrator-project de ontsluiting van online lotgenootverhalen te verbeteren voor borstkankerpatiëntes die erin geïnteresseerd zijn.

Waarom geen andere inclusiecriteria? Borstkankerpatiëntes die geïnteresseerd zijn in lotgenootverhalen hebben uiteenlopende persoonskenmerken: oud en jong, diagnose kort of lang geleden, nog onder behandeling of de behandelingen al achter de rug, enzovoorts. Het is dus onnodig om daar inclusiecriteria voor op te stellen. Bovendien staan dergelijke inclusiecriteria generalisatie mogelijk in de weg. Wij willen voor een zo divers mogelijke groep weten wat het effect is van het met of zonder zoekmogelijkheden aanbieden van lotgenootverhalen online.

Werving

Voor dit onderzoek zijn vrij veel deelneemsters nodig: zeker 25 per groep, wat neerkomt op in totaal 100 deelneemsters. Liever hebben we 200 deelneemsters, want er zit waarschijnlijk behoorlijke variatie in de belangrijkste afhankelijke variabele. Het precieze aantal deelneemsters kunnen we niet uitrekenen met een powerberekening, omdat we geen gevalideerde vragenlijst gebruiken. Om het grote aantal deelneemsters te bereiken, is het zinvol om via diverse wegen te werven:

1. Online, via diverse websites, zoals www.de-amazones.nl, www.borstkanker.nl, www.kwfkankerbestrijding.nl, www.kanker.info, www.kankerpatient.nl, www.ikcnet.nl.
2. Via flyers, in wachtkamers van het LUMC (bv. bij oncologie, of de mammapoli), en bij praatgroepen of inloopochtenden voor borstkankerpatiënten.

Op de websites wordt een button geplaatst met tekst in de trant van: “Bent u vrouw, heeft u borstkanker (gehad), en wilt u meedoen aan onderzoek naar de toegankelijkheid van online lotgenootverhalen? Klik dan hier.” Iemand die op deze button klikt, wordt naar de onderzoekssite doorgestuurd. Waarom werken we met een button en niet met een pop-up schermpje? Een pop-up schermpje stoort iemand die aan het internetten is. Het is een vrij irritante manier om de aandacht te vragen. Een button is veel netter. Iemand kan zelf beslissen of hij/zij de button aanklikt. Een nadeel is dat een button over het hoofd gezien kan worden, daarom moet de button op een vrij prominente plaats op de website(s) staan. Op de flyers wordt de URL van de onderzoekssite vermeld, zodat geïnteresseerden er zelf naartoe kunnen surfen.

Via de online werving zullen we waarschijnlijk deelneemsters krijgen met veel internetervaring die de verschillende websites met lotgenootverhalen al goed kennen. Via de

flyers zullen zich ook vrouwen aanmelden die minder ervaring hebben met internet en de websites met lotgenootverhalen niet kennen. Leidt dit tot een vertekening van de resultaten? Je zou kunnen beredeneren dat iemand die de ervaringsverhalen op de website van De Amazones al kent makkelijker zijn weg kan vinden dan iemand die ze nog niet kent, en dat daardoor een betere beoordeling gegeven wordt. Echter, doordat we randomiseren worden de twee soorten deelneemsters gelijkmatig over de vier onderzoeksgroepen verdeeld, dus zal vertekening beperkt blijven. Wel dient aan deelneemsters gevraagd te worden of ze internetervaring hebben, en of ze (de ervaringsverhalen op) de website van De Amazones kennen, zodat er in de analyse eventueel voor gecorrigeerd kan worden.

Op de onderzoekssite zal informatie over het onderzoek staan die kan worden uitgeprint. Deze informatiepagina eindigt met twee knoppen: “ik wil nu niet meedoen, wellicht op een later tijdstip” en “ik wil nu meedoen”. Vrouwen die de bedenktijd-knop aanklikken, kunnen op een later tijdstip - als ze besloten hebben toch mee te doen - weer naar de onderzoekssite surfen en dan “ik wil nu meedoen” aanklikken. Aan vrouwen die “ik wil nu meedoen” aanklikken wordt middels een online vragenlijstje gevraagd of ze vrouw zijn, of ze borstkanker hebben of hebben gehad, en of ze geïnteresseerd zijn in lotgenootverhalen. Degenen die al deze vragen met ‘ja’ beantwoorden, krijgen een *informed consent* voorgelegd. Vrouwen die akkoord gaan met het informed consent worden random toegewezen aan één van de vier groepen, en krijgen een inlognaam en wachtwoord. De “ik wil nu meedoen”-vrouwen dienen het onderzoek in één keer af te ronden, omdat ze anders door externe factoren beïnvloed zouden kunnen worden. Het is daarom belangrijk dat ze van tevoren gewaarschuwd worden dat het onderzoek enige tijd in beslag kan nemen.

Er kleven twee belangrijke nadelen aan het online werven en het online uitvoeren van het onderzoek. Ten eerste kunnen we niet checken of een bezoeker de drie vragen naar waarheid beantwoordt, met het gevolg dat we personen zouden kunnen includeren die niet aan de inclusiecriteria voldoen, waardoor de resultaten vertekend worden. Hier is geen oplossing voor. Echter, de kans dat een ‘grappenmaker’ mee gaat doen aan het onderzoek lijkt gering. Ten tweede zou een persoon die reeds mee heeft gedaan zich nog een keer kunnen aanmelden, wat ook kan leiden tot een vertekening van de resultaten. Ook hier is geen oplossing voor. Het enige wat je kan zien is of een aanmelding van dezelfde computer komt of niet. Door iedereen een andere inlognaam en wachtwoord te geven, kunnen we wel checken of vrouwen vaker hebben ingelogd en vaker de nameting vragenlijst hebben ingevuld.

Onderzoeksgroepen

Wat wordt van de deelneemsters gevraagd?

In elk van de vier groepen (de controlegroep en de drie interventiegroepen) wordt aan deelneemsters gevraagd om op zoek te gaan naar hun eigen informatiebehoefte in de set verhalen. Informatiebehoefte verschilt van persoon tot persoon: sommige informatiebehoeftes zullen wel bevredigd kunnen worden met de set verhalen en andere niet. Randomiseren zal ervoor zorgen dat de diverse informatiebehoeftes gelijkmatig over de vier groepen verdeeld worden. Door in de voormeting naar informatiebehoefte te vragen,

kan gecheckt worden of de verdeling inderdaad gelijkmatig is over de vier groepen, en kan er indien nodig voor gecorrigeerd worden in de analyse. Bovendien kan bekeken worden voor hoeveel deelneemsters de infobehoeftes niet bevredigd kunnen worden, omdat de verhalen zich daar niet voor lenen.

Waarom laten we de deelneemsters geen vooraf door de onderzoekers gedefinieerde taak uitvoeren die voor iedereen hetzelfde is, om controle te hebben over dit aspect? Ten eerste is dit ethisch gezien niet correct. Stel dat een patiënte niks over borstamputatie wil weten, omdat zij daardoor angstig wordt. En stel dat een onderdeel van de taak is om iets over amputatie op te zoeken. Dan wordt de deelneemster ‘gedwongen’ op zoek te gaan naar iets waar ze angstig van wordt. Dit laatste mag niet gebeuren. Ten tweede zullen deelneemsters veel meer geïnteresseerd zijn in hun eigen informatiebehoefte, en deelneemsters zullen voor de eigen informatiebehoefte ook veel beter kunnen aangeven of een bepaald zoekresultaat relevant voor ze is of niet.

Controlegroep

In Groep A, de controlegroep, worden de verhalen alfabetisch op naam van de schrijfsters aangeboden (figuur 2). Dit is de manier waarop de ervaringsverhalen momenteel op de website van Stichting Amazones worden aangeboden. Als een mapje wordt aangeklikt, verschijnt een rijtje met namen met de betreffende beginletter. Als vervolgens een naam wordt aangeklikt, wordt het hele ervaringsverhaal (vanaf het begin) getoond.

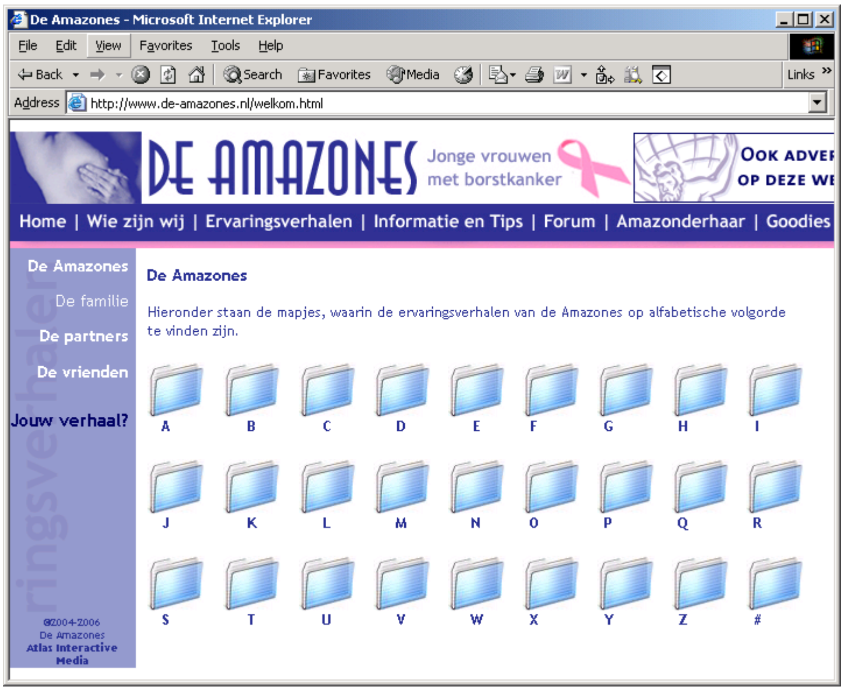

Figuur 2: Print screen van http://www.de-amazones.nl -> Ervaringsverhalen -> De Amazones.

Interventiegroepen

De drie interventiegroepen krijgen dezelfde verhalen aangeboden als de controlegroep. Ook de lay-out van de website wordt voor de interventiegroepen zoveel mogelijk gelijk

gehouden aan die van de controlegroep. Het enige verschil met de controlegroep is dat de verhalen anders toegankelijk gemaakt worden. In groep B worden de verhalen aangeboden middels een zoekmogelijkheid naar inhoud, in groep C middels een zoekmogelijkheid naar persoonskenmerken van schrijfsters, en in groep D middels zowel een zoekmogelijkheid naar inhoud als naar persoonskenmerken van schrijfsters (figuur 3).

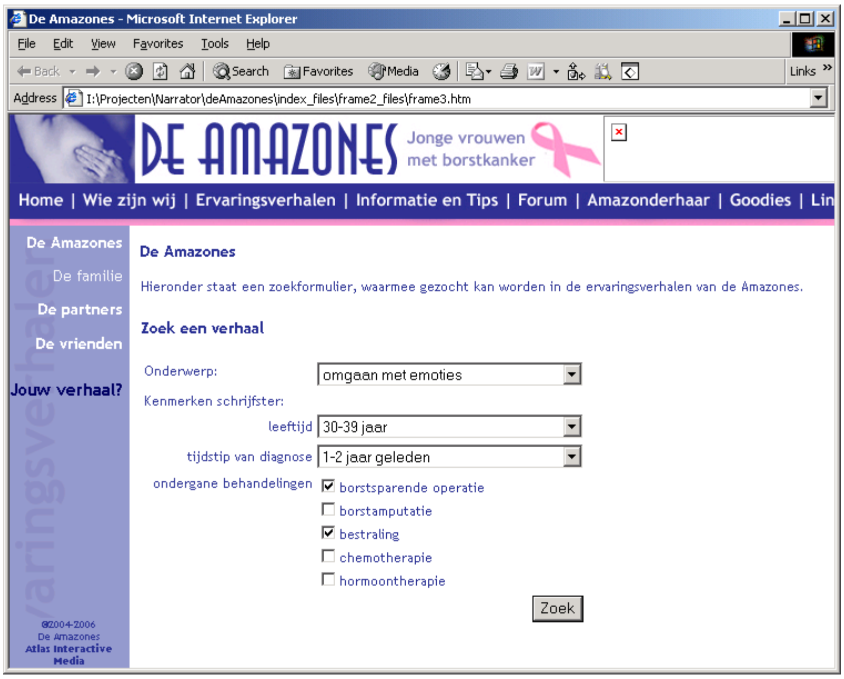

Figuur 3: Voorbeeld van hoe het zoekformulier er voor groep D uit zou kunnen zien. (Let op!: dit is een voorbeeld om het idee duidelijk te maken. Het is geen print screen van de website van Stichting Amazones.)

Hoe worden alle opgehaalde verhalen in zijn totaliteit gepresenteerd na een zoekactie? Er zijn twee mogelijkheden: op relevantie of op alfabet. Om de verhalen op relevantie te presenteren, moeten er criteria voor opgesteld worden. Dit is een vrij lastige klus, die zeker wat tijd gaat kosten. Voordelen van het op relevantie presenteren is dat het wellicht aantrekkelijker is voor de gebruiker en dat je ook verhalen in de lijst kunt opnemen die niet helemaal aan het gezochte voldoen, maar wel bijna. Op alfabet presenteren is snel en makkelijk. Een groot nadeel van op alfabet presenteren is dat je alleen die verhalen kan tonen die precies kloppen met het gezochte. Op relevantie presenteren geniet daarom toch de voorkeur, ook omdat dan het verschil tussen de interventiegroepen en de controlegroep gemaximaliseerd wordt.

Hoe wordt elk verhaal afzonderlijk gepresenteerd na een zoekactie? Wederom zijn er meerdere mogelijkheden. Een verhaal zou gepresenteerd kunnen worden door alleen de auteursnaam te vermelden, of alleen de titel of een combinatie van deze twee. Echter, deze manier van presenteren biedt geen aanvullende informatie aan de gebruiker waarmee hij/zij kan beslissen welk verhaal hij/zij het eerste aanklikt. Een andere mogelijkheid is om elk verhaal op een Google-achtige wijze weer te geven, waarbij al wat context getoond wordt voordat het verhaal is aangeklikt. Het is vrij lastig dit laatste voor elkaar te krijgen, omdat er handmatig anchors moeten worden aangebracht in de

verhalen. Toch lijkt Google-achtige presentatie de beste optie, omdat het wederom het verschil tussen de interventiegroepen en de controlegroep vergroot.

Hoe wordt binnen elk verhaal de zoekactie gepresenteerd? Persoonskenmerken worden aan het begin van het verhaal kort en puntsgewijs in een lijstje vermeld. De persoons-kenmerken waarop gezocht is, worden -indien mogelijk- gehighlight. Ook de delen van het verhaal die gaan over de gezochte inhoud worden -indien mogelijk- gehighlight. De relevante delen van het verhaal zouden ook met anchors aangegeven kunnen worden, maar als je op meerdere onderwerpen tegelijk zoekt, is dat wat lastiger. Door middel van het highlighten wordt tegemoet gekomen aan één van de interviewresultaten, te weten: dat vrouwen liefst korte tekstfragmenten over een bepaald onderwerp lezen.

Hoe wordt het zoeksysteem voor de interventiegroepen gerealiseerd?

Om het zoeksysteem voor de interventiegroepen te realiseren zijn twee stappen nodig: 1) het toekennen van metadata aan de verhalen; en, 2) met behulp van de gegevens uit de eerste stap het zoeksysteem daadwerkelijk bouwen (programmeren)

- Metadata toekennen aan de verhalen

Persoonskenmerken. In groep C en D kunnen deelneemsters verhalen selecteren op een aantal persoonskenmerken van schrijfsters, te weten: geboortejaar, relatie/burgerlijke staat, wel/geen kinderen, jaar waarin diagnose is gesteld, ondergane behandelingen, en wel/geen uitzaaiingen. De eerste vijf zijn voor de meeste verhalen op de website van De Amazones te vinden (onder ‘Wie zijn wij’, en vervolgens ‘De Amazones’). Als ze daar niet te vinden zijn, kan uiteraard gekeken worden of ze uit het verhaal zelf zijn af te leiden. Voor het laatste kenmerk ‘wel/geen uitzaaiingen’ moet ook in de verhalen zelf gekeken worden.

Waarom deze persoonskenmerken? In het kader van het Narrator-project zijn reeds interviews afgenomen bij borstkankerpatiëntes waarin onder meer gevraagd is op basis van welke persoonskenmerken van schrijfsters ze verhalen willen selecteren. Meer dan de helft van de ondervraagden wilde op de bovenstaande persoonskenmerken kunnen selecteren. Een uitzondering hierop vormt ‘relatie/burgerlijke staat’, daarop wilde iets minder dan de helft van de deelneemsters kunnen selecteren. Waarom wordt dit kenmerk dan toch meegenomen in het onderzoek? De ‘relatie/burgerlijke staat’ is voor de meeste van de schrijfsters bekend; het zou daarom onvolledig zijn dit persoonsken-merk niet mee te nemen in dit onderzoek.

Wat te doen met eventuele missende persoonskenmerken? Het zou kunnen dat één of meer van de zes persoonskenmerken niet bekend is voor sommige schrijfsters. Het is niet nodig om in dat geval het hele verhaal uit te sluiten; het is voldoende een missing te noteren voor het ontbrekende persoonskenmerk. Als iemand bijvoorbeeld alleen op geboortejaar wil zoeken, dan maakt het niet uit als de burgerlijke staat van de schrijfster onbekend is. Wel is het belangrijk na het toekennen van de metadata te kijken hoeveel verhalen er compleet zijn wat betreft persoonskenmerken, en hoeveel niet. Als er relatief veel verhalen incompleet zijn, zou dit een verstorende factor (covariaat) kunnen worden die de uitkomst ongewenst beïnvloed.

Wie gaat persoonskenmerken toekennen aan de verhalen, en hoe? RO kent de per-soonskenmerken toe aan de verhalen. Omdat het om feitelijke informatie gaat, die niet multi-interpretabel is, is het niet nodig het toekennen door een 2^{e} persoon te laten

controleren. De gegevens zullen in MS Excel, MS Access, of SPSS gezet worden, afhankelijk van wat voor AdM, die het zoeksysteem gaat bouwen, het handigst is.

Inhoud. In groep B en D willen we het mogelijk maken dat deelneemsters op inhoud van verhalen kunnen zoeken. Hiertoe is het nodig om codes toe te kennen voor de onderwerpen die voorkomen in de verhalen.

Welke onderwerpen? Wellicht is de beste optie om gezamenlijk te komen tot een coderingssysteem. Hierbij kan gebruikt gemaakt worden van de resultaten van de interviews die in het kader van het Narrator-project reeds zijn afgenomen bij borstkankerpatiëntes en van frameworks uit de literatuur. Als het coderingssysteem af is, kan gekeken worden hoe betrouwbaar twee codeurs ermee kunnen werken.

Wie gaat de codes voor de onderwerpen toekennen aan de verhalen, en hoe? De codes zullen worden toegekend door twee beoordelaars, onafhankelijk van elkaar, te weten RO en PT. Waarom? De inhoud van verhalen is veelal op meerdere manieren te interpreteren, waardoor er kans bestaat op verkeerd toekennen van metadata. Als iemand zoekt op onderwerp A en er wordt een verhaal getoond dat niet over onderwerp A gaat, zal deze deelneemster wellicht niet tevreden zijn en het getoonde verhaal niet kunnen gebruiken, terwijl zij het hebben van de zoekmogelijkheid wel op prijs zou stellen als deze goed zou werken. Door twee personen de inhoud te laten beoordelen, is de kans op verkeerd toekennen van codes kleiner. Deze manier is dus betrouwbaarder dan de codes laten toekennen door slechts één persoon. De verhalen zullen worden geïmporteerd in NVivo, waarin PT en RO elk een eigen project kunnen aanmaken om de verhalen te coderen. Nadat beide beoordelaars de verhalen hebben gecodeerd kan gekeken worden in hoeverre de coderingen overeenkomen.

- Het bouwen van het zoeksysteem

Met gebruikmaking van inhoudelijke input van RO, zal AdM het zoeksysteem gaan bouwen. De gevonden verhalen worden na een zoekactie gepresenteerd op relevantie, met per verhaal reeds wat context. Indien mogelijk worden de kenmerken waarop gezocht is gehighlight in de gevonden verhalen.

Persoonskenmerken. Per persoonskenmerk zal de zoekmogelijkheid enigszins verschillen (zie tabel 1).

Inhoud. Het zoeken naar inhoud zal mogelijk gemaakt worden door middel van een afrolmenu, waarbij naar onderwerpen die in de lijst voorkomen gezocht kan worden. Het is mogelijk naar één of naar meerdere onderwerpen tegelijk te zoeken. Veel mensen weten niet dat je met een afrolmenu ook naar meerdere onderwerpen tegelijk kan zoeken, dus dit moet duidelijk uitgelegd worden aan de deelneemsters.

Waarom zal het niet mogelijk zijn om met behulp van eigen gekozen zoektermen (vrije tekst) te zoeken? De technieken op dit gebied zijn nog niet geavanceerd genoeg. Met behulp van vrije tekst kan momenteel alleen nog maar gezocht worden naar woorden die letterlijk in een verhaal voorkomen, en hierin zijn we niet geïnteresseerd, we willen naar inhoud zoeken. Een voorbeeld. Stel je zoekt met vrije tekst naar 'radiotherapie', dan zul je alleen verhalen getoond krijgen waarin het woord radiotherapie letterlijk voorkomt. Verhalen waarin het woord 'bestraling' voorkomt, zullen niet getoond worden, terwijl bestraling en radiotherapie hetzelfde betekenen. Je moet dan eigenlijk een programma hebben dat 'weet' dat bestraling hetzelfde betekent als radiotherapie, zodat je, als je op radiotherapie zoekt, ook de verhalen over bestraling getoond krijgt. Maar

zo'n programma is voor zover wij weten niet voorhanden, en daarom wordt er met een afrolmenu en vooraf gedefinieerde zoektermen gewerkt.

Tabel 1. Zoekmogelijkheden per persoonskenmerk.

Persoonskenmerken	*Zoekmogelijkheid*
geboortejaar	afrolmenu; ranges (bv. 1950-1955, 1956-1960) + optie 'geen voorkeur'
relatie/burgerlijke staat	afrolmenu; enkele opties (bv. samenwonend, alleenstaand) + optie 'geen voorkeur'
wel/geen kinderen	afrolmenu; twee opties (wel kinderen, geen kinderen) + optie 'geen voorkeur'
jaar waarin diagnose is gesteld	afrolmenu; ranges + optie 'geen voorkeur'
ondergane behandelingen	aanklikken; zes behandelingen waarvan één of meerdere kunnen worden aangeklikt (borstsparende operatie; borstamputatie; bestraling; chemotherapie; hormoontherapie; immunotherapie)
wel/geen uitzaaiingen	afrolmenu; twee opties (wel uitzaaiingen, geen uitzaaiingen) + optie 'geen voorkeur'

Randomisatie en inlogprocedures

De randomisatieprocedure zal zo zijn dat begonnen wordt met het vullen van de groepen A en C. Waarom? De interventie voor groep A bestaat al (dat is immers de huidige website) en de interventie voor groep C is redelijk snel en makkelijk te maken. Door te starten met de groepen A en C kunnen we kijken of alles werkt zoals we verwachten: of de techniek goed werkt, of zich genoeg deelneemsters aanmelden, enzovoorts. Een nadeel van deze methode is dat er een extra factor geïntroduceerd wordt: tijd. Mensen die zich in het begin aanmelden hebben een grote kans om in groep A of C terecht te komen, mensen die zich later aanmelden hebben meer kans om in groep B of D geplaatst te worden. Echter, wij verwachten niet dat deze extra factor 'tijd' invloed zal hebben op het resultaat. Het zou wel kunnen dat het aantal aanmeldingen in oktober een piek vertoond, omdat dan vanwege de borstkankermaand veel aandacht voor borstkanker in de media zal zijn.

Hoe gaan we randomiseren? Indien mogelijk laten we de 'technische taal' achter de "ik wil nu meedoen"-knop een willekeurig getal genereren tussen 0-1. Het gegenereerde getal bepaalt in welke groep iemand terecht komt. Gedurende het eerste deel van het onderzoek worden deelneemsters alleen in groep A (0-0.50) en C (0.51-1) geplaatst. Als het goed loopt, en we verwachten voldoende deelneemsters te krijgen voor alle groepen, gaan we door met werven. Gedurende dit tweede deel van het onderzoek zullen deelneemsters alleen in groep B (0-0.50) en D (0.51-1) geplaatst worden. Als de nog beschikbare tijd en het aantal aanmeldingen het toelaten, kan op een gegeven moment

besloten worden om deelneemsters te plaatsen in alle vier groepen: A (0-0.25), B (0.26-0.50), C (0.51-0.75) en D (0.76-1).

De verhalen met zoekmogelijkheden zijn tijdens het onderzoek alleen bedoeld voor de deelneemsters die ingedeeld zijn in één van de interventiegroepen. Met behulp van een inlogprocedure wordt ervoor gezorgd dat alleen zij erbij kunnen.

Ook voor de controlegroep is het wellicht handig een inlogprocedure te hanteren. Ten eerste is dat voor de log-gegevens handig: je weet dan precies wanneer iemand inlogt en begint aan het onderzoek. Ten tweede zou dan de site voor de controlegroep ‘bevroren’ kunnen worden. Als er tijdens het onderzoek nieuwe verhalen worden aangeleverd, worden die wel op de openbare website gezet, maar niet op de ‘bevroren’ website voor de controlegroep. Dit laatste zorgt ervoor dat de controlegroep ten allen tijde precies dezelfde set verhalen aangeboden krijgt als de interventiegroepen.

Metingen

Waar vinden de metingen plaats?

De metingen vinden online plaats. Deelneemsters kunnen meedoen aan het onderzoek terwijl ze thuis achter hun eigen computer zitten. Waarom kiezen we hiervoor? Ten eerste krijgen we dan waarschijnlijk meer deelneemsters dan als we ze naar het LUMC laten komen, omdat het deelneemsters minder energie en tijd kost als ze niet hoeven reizen. Ten tweede is het realistisch: in het ‘echte leven’ surfen deelneemsters ook vanuit hun eigen omgeving op het internet. Er zijn wel enkele nadelen. De kans bestaat dat deelneemsters beïnvloed worden door eventuele derden. Echter, het randomiseren zorgt ervoor dat de door derden beïnvloedde deelneemsters gelijkmatig over de vier groepen verdeeld worden. Ten tweede kunnen zich technische storingen voordoen. Daarom moet er een telefoonnummer beschikbaar zijn dat de deelneemsters kunnen bellen bij technische storingen. Tot slot, en dit is wellicht het grootste nadeel, weet je online nooit met wie je te maken hebt: zijn het wel echt vrouwelijke borstkankerpatiënten en hoe weten we dat iemand niet vaker dan één keer meedoet aan het onderzoek.

Voormeting

In de voormeting wordt één open vraag over informatiebehoefte gesteld. Waarom zo’n korte voormeting? Een vragenlijst vooraf is eigenlijk ook al een soort interventie waardoor deelneemsters beïnvloed worden. Door de vragenlijst vooraf zo kort mogelijk te houden, worden deelneemsters zo min mogelijk beïnvloed. Om dezelfde reden wordt er een open vraag gesteld, en wordt er geen lijstje gegeven met onderwerpen en persoonskenmerken die deelneemsters kunnen aankruisen. Door een open vraag te stellen worden deelneemsters niet beïnvloed door informatie die wij aandragen. De vraag zal er ongeveer als volgt uitzien: “Voordat u gaat zoeken in de lotgenootverhalen zouden we u graag één vraag willen stellen. Waar bent u naar op zoek?” De antwoorden op deze voormeting zullen onder meer gecodeerd worden op compleetheid en duidelijkheid.

Meting tijdens interventie

Tijdens de interventie worden log-gegevens bijgehouden. Daarmee kan voor elke deelneemster berekend worden hoe lang ze ingelogd is geweest (wat overigens niet hoeft te betekenen dat ze gedurende die hele tijd bezig is geweest met het zoeken in en lezen van de ervaringsverhalen). Voor groep A kan gekeken worden welke beginletters van auteursnamen en welke verhalen zijn aangeklikt, voor groep B op welke onderwerpen is gezocht en of er ook naar meerdere onderwerpen tegelijk is gezocht, en voor groep C op welke persoonskenmerken is gezocht en of er ook naar meerdere persoonskenmerken tegelijk is gezocht. Tot slot kan voor groep D gekeken worden of deelneemsters alleen op inhoud hebben gezocht, of alleen op persoonskenmerken, of dat ze ook zoekacties gedaan hebben waarbij inhoud en persoonskenmerken zijn gecombineerd. Het is ook interessant te kijken hoeveel zoekacties door de deelneemsters zijn uitgevoerd, en de verschillen tussen de opeenvolgende zoekacties. Scherpt een deelneemster haar zoekactie steeds iets meer aan, of verschillen zoekacties geheel van elkaar?

Verder kunnen de log-gegevens informatie verschaffen over hoe deelneemsters hun informatiebehoefte naar de praktijk vertaald hebben. Dit kan gedaan worden door het antwoord op de open vraag naar informatiebehoefte in de voormeting te vergelijken met wat deelneemsters daadwerkelijk gezocht hebben.

Tot slot kunnen de log-gegevens gebruikt worden om te kijken of deelneemsters verhalen zoeken van vergelijkbare lotgenoten, door te kijken of de persoonskenmerken van de deelneemsters zelf (wordt naar gevraagd in de nameting) overeenkomen met de persoonskenmerken waarop gezocht wordt.

Nameting

In de nameting worden covariaten, de uitkomstmaten, en nogmaals de inclusiecriteria nagevraagd. Dit laatste wordt gedaan als een extra check. Stel er zijn toch mensen geïncludeerd die niet aan de inclusiecriteria voldoen, dan kunnen zij er alsnog uitgehaald worden, zodat hun gegevens niet meegenomen worden in de analyse.

Covariaten zijn variabelen die de uitkomstmaten van het onderzoek kunnen beïnvloeden. In de analyse dient voor de mogelijke covariaten gecorrigeerd te worden. Mogelijke <u>covariaten</u> in dit onderzoek, die zullen worden nagevraagd in de nameting, zijn:

- demografische kenmerken (zoals leeftijd, burgerlijke staat, wel/geen kinderen, opleidingsniveau);
- ziektekenmerken (zoals tijd sinds diagnose, soort borstkanker, moment in het ziektebeloop, ondergane en toekomstige behandelingen, wel/geen uitzaaiingen);
- internetervaring (sinds wanneer gebruikt men internet, hoe frequent, wat doet men zoal op internet, vaker naar lotgenootverhalen gezocht, welke websites bezocht);
- bekend met de website van De Amazones (kent men de website, de ervaringsverhalen, het forum; is men zelf een Amazone, schrijfster van één van de verhalen);
- eerder lotgenootverhalen gezocht/gelezen/gehoord via praatgroepen/boeken.

De <u>uitkomstmaten</u> van dit onderzoek zijn: 1) tevredenheid met het zoekproces, en 2) bruikbaarheid van de gevonden verhalen. Elk van deze twee categorieën is weer verder in te delen. Zo valt <u>tevredenheid met het zoekproces</u> uiteen in:

- mening over het soort en het aantal zoekmogelijkheden;

- mening over het voldoen van de zoekresultaten aan de verwachting van de zoekactie;
- meer/andere zoekmogelijkheden gewenst;
- enzovoorts.

De bruikbaarheid van de gevonden verhalen kan opgesplitst worden in een aantal aspecten. Bijvoorbeeld, zijn de gevonden verhalen bruikbaar voor:

- herkenning/verbonden voelen/het gevoel niet de enige te zijn/weten dat anderen het ook moeilijk hebben;
- kennis/informatie/keuze behandeling/geïnformeerd zijn om artsen de goede vragen te kunnen stellen;
- leren omgaan met de ziekte;
- (sociale) steun/verwerking/hulp/het zelf een plek geven;
- jezelf voorbereiden/weten wat je te wachten staat;
- eigen plaats bepalen/bevestiging krijgen/erkenning/is het normaal wat ik voel;
- praktische tips;
- positieve voorbeelden/verhalen met goede afloop;
- mening over de relevantie en nuttigheid van de gevonden verhalen;
- inzicht krijgen in de eigen problemen;
- toepasbaarheid op de eigen situatie;
- perceived control;
- coping met de ziekte;
- repareren eigen illness story.

Vanwege het feit dat de nameting online afgenomen wordt, en dat mensen vaak geen zin hebben om lange vragenlijsten op de computer in te vullen, moet de nameting zo kort mogelijk zijn. Verder moet het deel van de vragenlijst over de uitkomstmaten heel gevoelig zijn, omdat het over zo'n specifiek onderwerp gaat: het zoekproces naar en de bruikbaarheid van lotgenootverhalen. Er wordt in de literatuur gekeken of er delen van gevalideerde vragenlijsten bruikbaar zijn voor dit onderzoek. De bruikbare delen worden aangevuld met eigen ontwikkelde vragen, die in een pilot getest worden bij collega onderzoekers en een aantal patiënten.

Techniek

AdM is verantwoordelijk voor de techniek in dit onderzoek. AdM verkent de technische mogelijkheden, zoekt eventueel technische oplossingen, en past de techniek toe om de wensen en inhoudelijke input van RO te realiseren. Onderdelen waarvoor gezorgd moet worden, zijn:

- deelname-knoppen op de website van De Amazones en op externe websites;
- pagina met informatie over het onderzoek, online vragenlijst om inclusiecriteria te checken en *informed consent*;
- randomisatieprocedure;
- inlogprocedure en log-gegevens;

- een goed werkend zoeksysteem voor de drie interventiegroepen (zoekformulieren; presentatie verhalen na een zoekactie; zichtbaarheid zoekactie binnen elk verhaal);
- online vragenlijsten (voor- en nameting).

Analyse en rapportage

De resultaten worden met behulp van SPSS geanalyseerd. Grofweg zal de analyse uit twee stappen bestaan, waarin waarschijnlijk de toetsen (M)ANOVA en (M)ANCOVA gebruikt moeten worden:

1. De baseline characteristics worden vergeleken tussen de controlegroep en de interventiegroepen.
2. De uitkomst van de nameting wordt vergeleken tussen de controlegroep en de interventiegroepen. (Als er een verschil is gevonden in baseline characteristics (bij 1), zal daarvoor in deze stap 2 gecorrigeerd worden).

Er wordt een wetenschappelijk artikel geschreven over het onderzoek, dat bij voorkeur wordt ingediend bij de British Medical Journal (impact factor= 7.038; http://bmj.bmjjournals.com/aboutsite/index.shtml). RO wordt eerste auteur, de andere betrokkenen worden medeauteurs of bedankt in de acknowledgements.

Planning

Totale studie: 11 maanden

Maand	*Activiteit*	*Wie?*
Mei, juni 2006	Plan uitwerken	RO, met hulp van WO en JV
Juli 2006	Bij Commissie Medische Ethiek van het LUMC vragen of beoordeling noodzakelijk is, en zo ja, indienen bij de CME	RO
Juli, augustus 2006	Vragenlijsten maken + gewenste log-gegevens inventariseren	RO, met hulp van WO en JV
	Persoonskenmerken schrijfsters toekennen aan verhalen + contacten leggen voor werving deelneemsters (knoppen op externe sites; en flyers, in wachtkamers, bij praatgroepen)	RO
	Website voor groep A en C gereed maken + deelname knoppen op externe sites	AdM
September, oktober 2006	Deelnemers werven starten + dataverzameling starten met groep A en C	RO
	Onderwerpen toekennen aan de verhalen	RO en PT
	Website voor groep B en D gereed maken	AdM

Maand	*Activiteit*	*Wie?*
November, december 2006	Deelnemers werven vervolgen + dataverzameling vervolgen met groep B en D	RO
Januari 2007	Dataverzameling voor alle vier groepen afsluiten	RO
Februari, maart 2007	Analyse en rapportage	RO, met hulp van WO en JV

Expertise, voorgaande activiteiten en producten

De groep Klinische informatiekunde (KIK) houdt zich bezig met de bijdragen die informatie- en communicatietechnologie kan leveren aan de klinische zorg en maakt gebruik van methoden en technieken van disciplines als de logica, informatica, taalwetenschappen, systeemtheorie, besliskunde, operations research, sociale wetenschappen, en bestuurs- en bedrijfskunde. Naast onderzoek en onderwijs heeft de Klinische Informatiekunde een adviesfunctie en participeert ze in innovatieve ICT-projecten in het Leids Universitair Medisch Centrum en de regio.

De centrale vraag in het onderzoek van de KIK is: welke communicatieprocessen komen voor in de gezondheidszorg en hoe beïnvloedt informatie- en communicatie-technologie (ICT) de communicatie en besluitvorming bij zorgverleners en patiënten. Vele vormen van ICT worden onderzocht: variërend van zorgwerkstations en Elektronische Patiëntendossiers tot dialooggestuurde websites.

Eén van de door KIK geïnitieerde onderzoeksprojecten is het Narrator-project. Dit project richt zich specifiek op patiënt-patiënt communicatie: het inwinnen van informatie door de patiënt bij (ex-)lotgenoten/medepatiënten over de (mogelijke) impact die de ziekte en de behandeling kan hebben op de kwaliteit van leven, en over wat hij/zij zelf kan doen om het ziektebeloop en de verwerking in positieve zin te beïn-vloeden. Er wordt onderzocht aan welke eisen een informatiesysteem zou moeten voldoen dat verhalen van lotgenoten ontsluit voor patiënten. De KIK doet ten behoeve van het Narrator-project onderzoek naar: a) lotgenootverhalen (waar gaan ze over, hoe zijn ze gestructureerd, hoe zou je ze automatisch kunnen clusteren, enzovoorts), b) de behoefte van patiënten (waarover willen zij lezen in lotgenootverhalen, hoe willen ze naar lotgenootverhalen zoeken in een informatiesysteem, hoe willen ze ze gepresenteerd krijgen, enzovoorts), en c) het koppelen van bestaande software om een innovatieve, synergetische werking te bewerkstelligen, die geschikt is voor het beoogde informatie-systeem. Twee externe onderzoeksgroepen zijn betrokken bij het Narrator-project: 1) de J.F. Schouten School for User-System Interaction Research (TU/e), en 2) Leiden University Centre for Linguistics (UL).

Het in dit document voorgestelde onderzoek zal als deelonderzoek binnen het Narrator-project worden uitgevoerd.

Eigen publicaties (gerelateerd aan het Narrator-project, op alfabet)

Alpay LL, Overberg RI, Zwetsloot-Schonk JHM. Empowering citizens in assessing health related websites: a driving factor for healthcare governance. *International Journal of Healthcare Technology and Management*, in press.

Hoenkamp E, Overberg R. Computing Latent Taxonomies from Patients' Spontaneous Self-Disclosure to Form Compatible Support Groups. (accepted for oral presentation at the MIE2006 conference on 27-30 August 2006).

Overberg R, Toussaint P, Zwetsloot-Schonk B. Illness stories on the Internet: Features of websites disclosing breast cancer patients' illness stories in the Dutch language. *Patient Education and Counseling*, in press.http://dx.doi.org/10.1016/j.pec.2005.05.010.

Overberg RI, Alpay LL, Verhoef J, Zwetsloot-Schonk JHM. Illness stories on the Internet: What do breast cancer patients want at the end of treatment? (submitted).

Overberg RI, Campagne AE, Zwetsloot-Schonk JHM. Empowering patients: Use and appreciation of a web application that enables patients to keep their own medical data and to share illness experiences with fellow patients. (accepted for poster presentation at the EACH 2006 conference on 5-8 September 2006).

Wolf L, Hoenkamp E, Overberg R, Reckman H, Toussaint P. Design of the Narrator system: processing, storing and retrieving medical narrative data. *SDPS Transactions-ISoLA*, in press.
