# Supplementary material for: How Breast Cancer Patients Want to Search for and Retrieve Information From Stories of Other Patients on the Internet: an Online Randomized Controlled Experiment
Source: J Med Internet Res. 2010 Mar 9;12(1):e7. doi: 10.2196/jmir.1215 (PMC2855205; doi:10.2196/jmir.1215)

Multimedia Appendix 1:

Screenshots illustrating the search page and the list of retrieved stories for each of the 4 search conditions (i.e., control, story topics, writer profile, and combination condition).

Appendix to:

Overberg R, Otten W, De Man A, Toussaint P, Westenbrink J, Zwetsloot-Schonk B. How breast cancer patients want to search for and retrieve information from stories of other patients on the Internet: an online randomised controlled experiment.

Screenshot 1a: The search page of the control group.

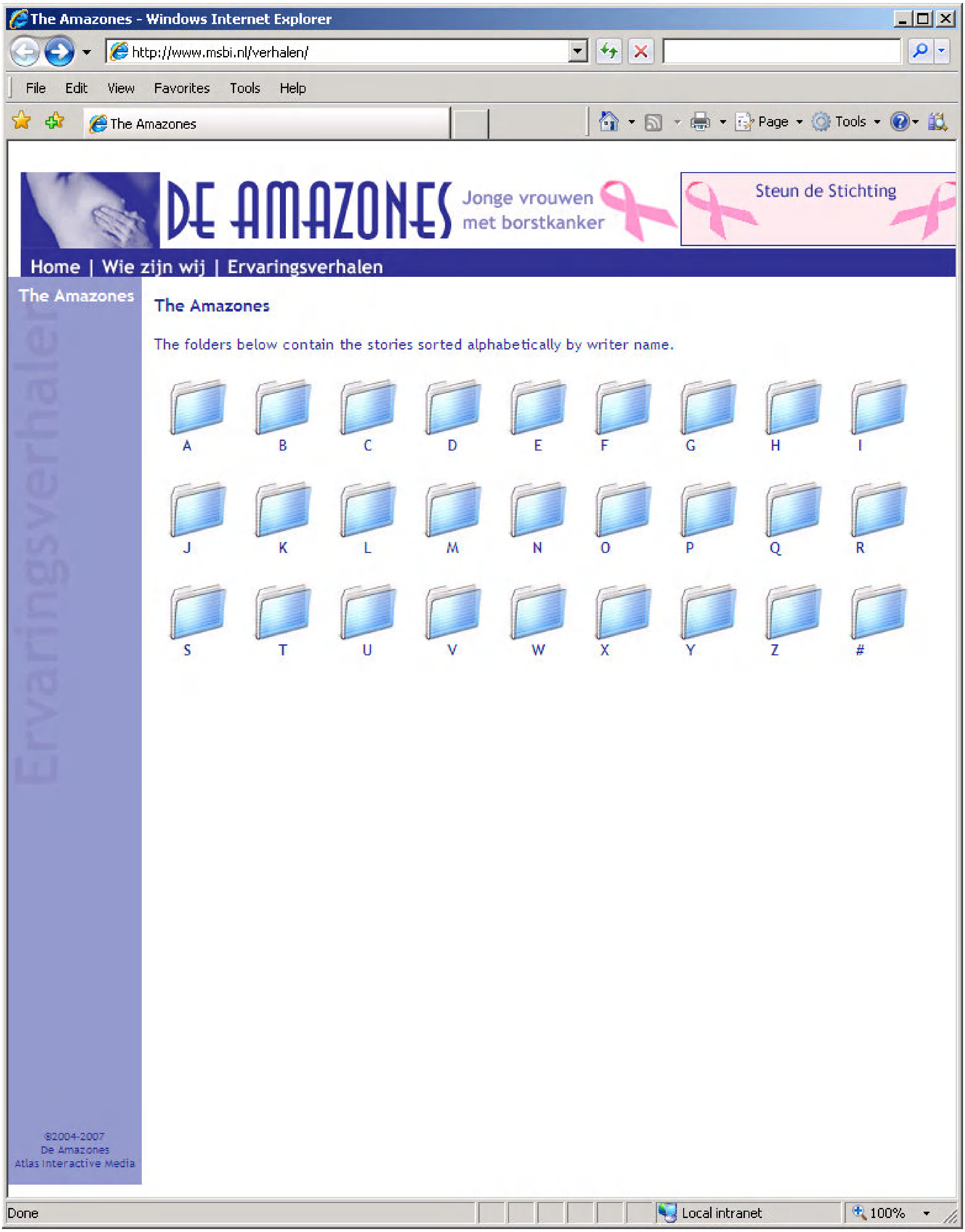

Screenshot 1b: A list of stories retrieved in the control group.

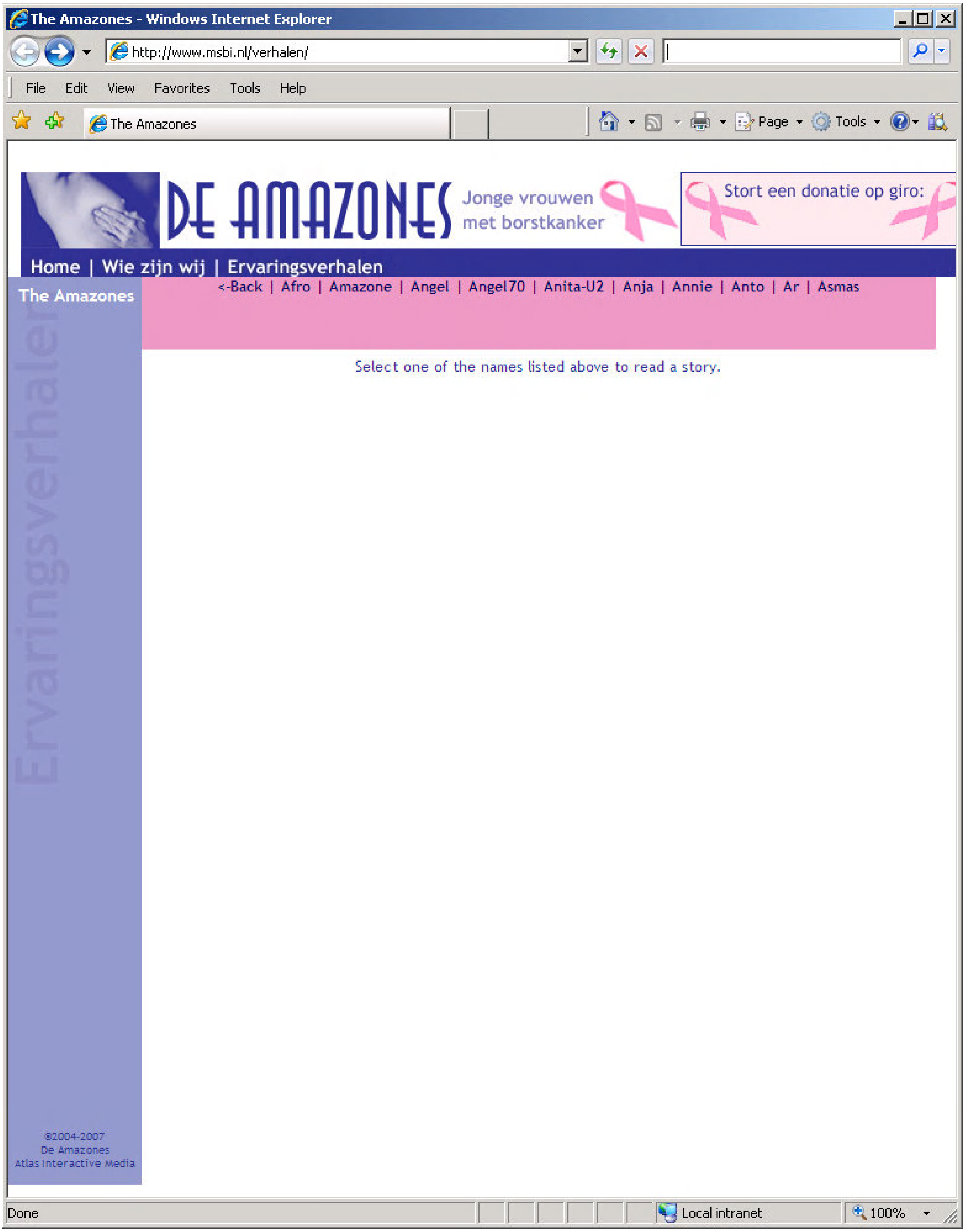

Screenshot 2a: The search page of the story topics group.

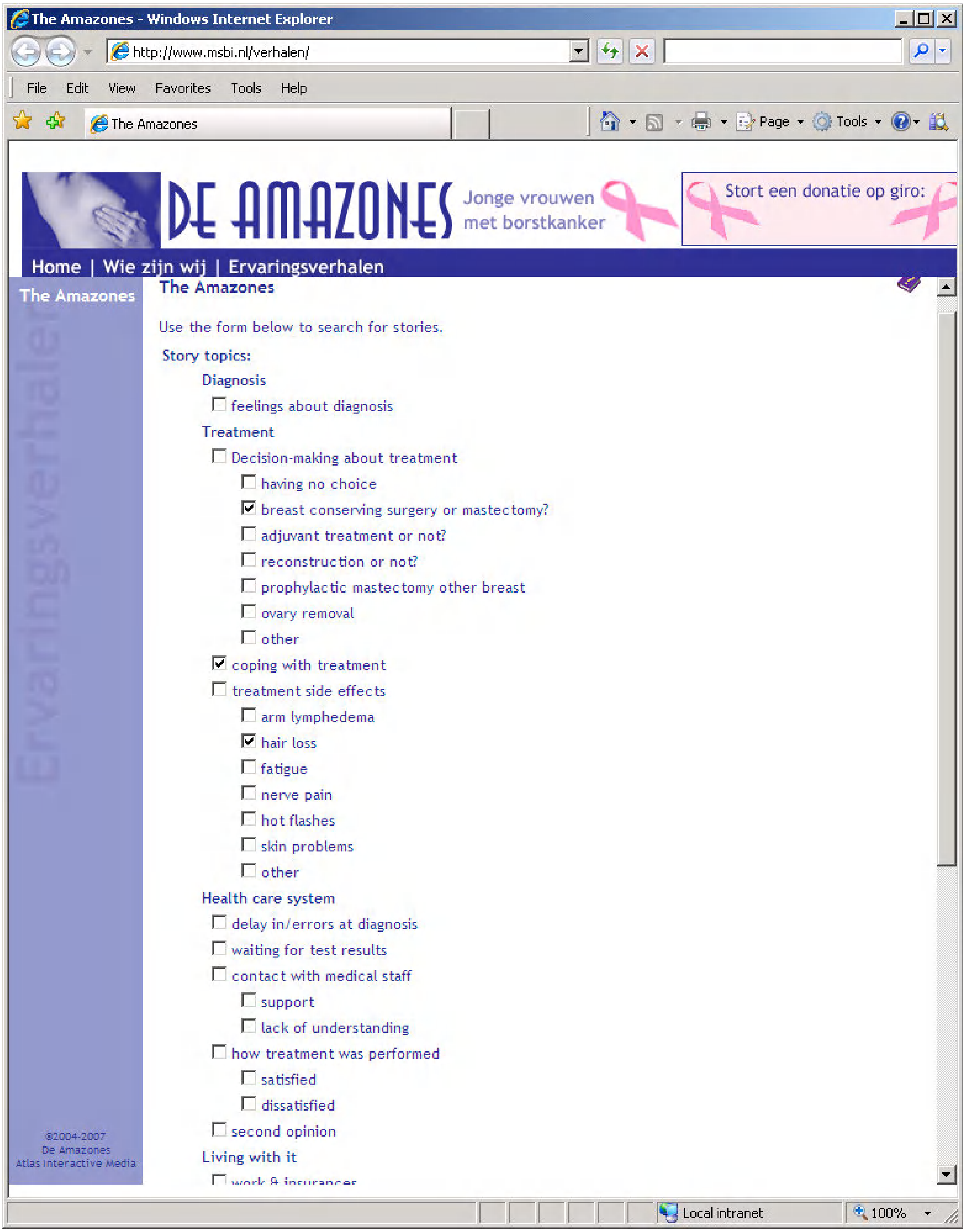

Screenshot 2b: A list of stories retrieved in the story topics group.

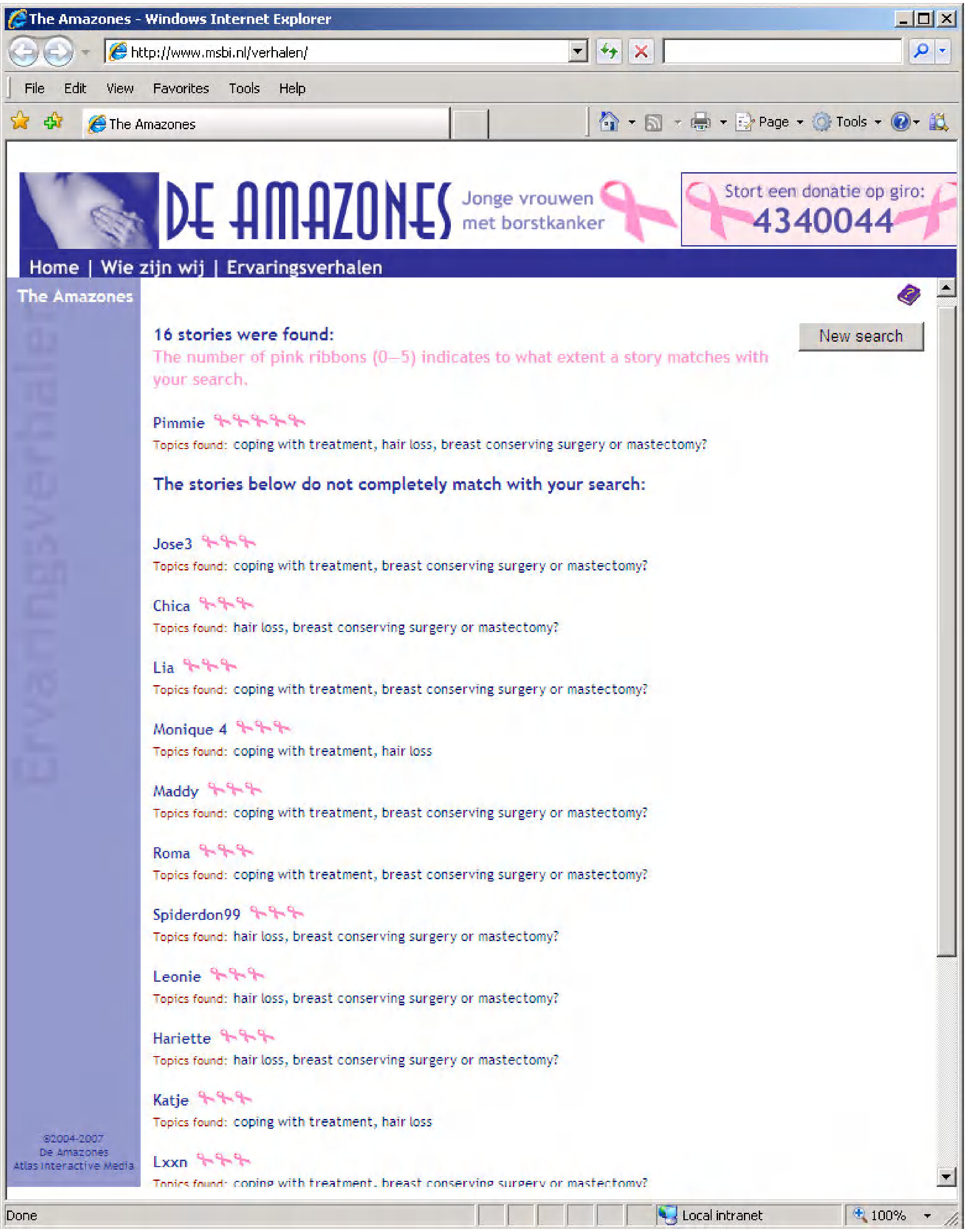

Screenshot 3a: The search page of the author profile group.

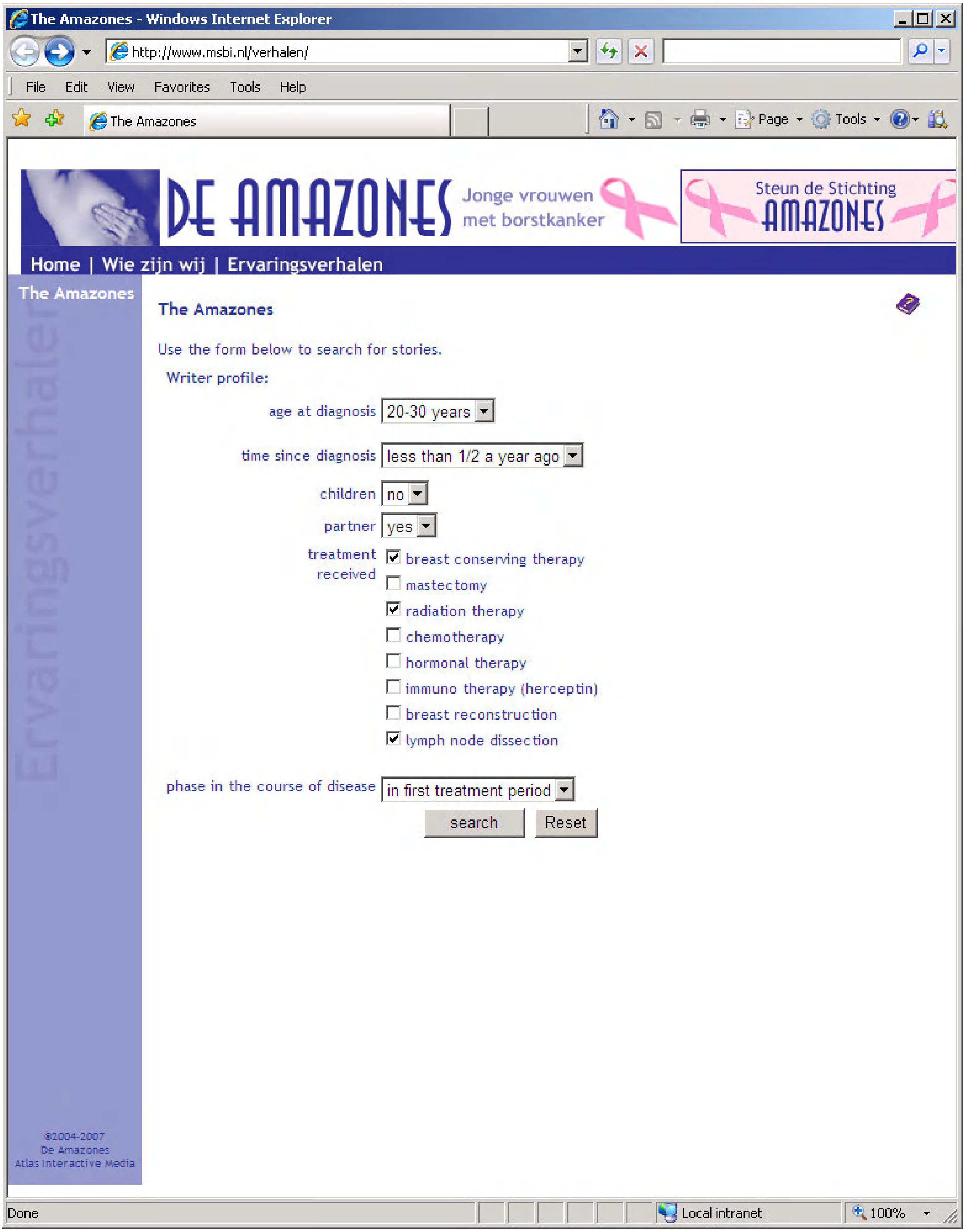

Screenshot 3b: A list of stories retrieved in the author profile group.

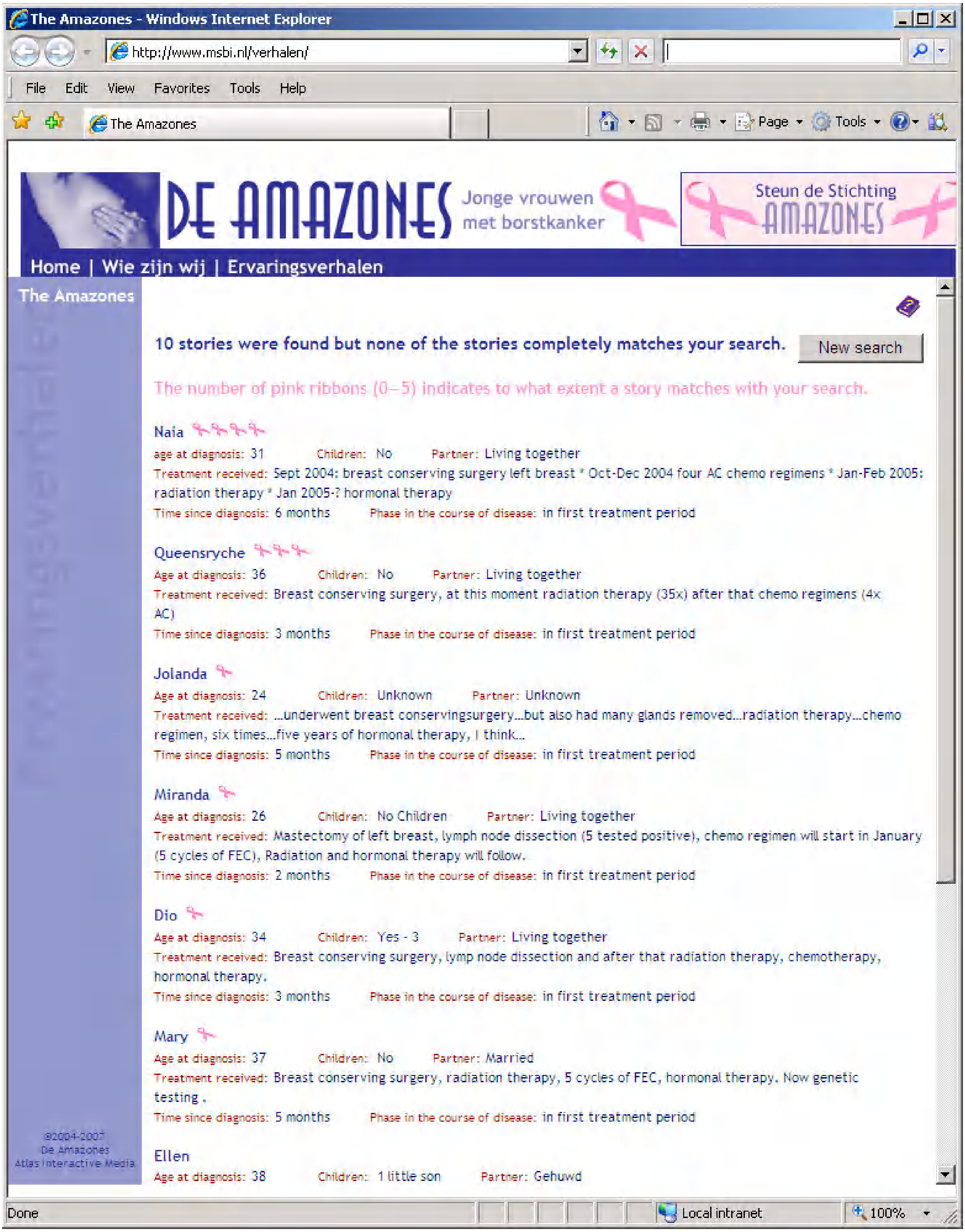

Screenshot 4a: The search page of the combination group.

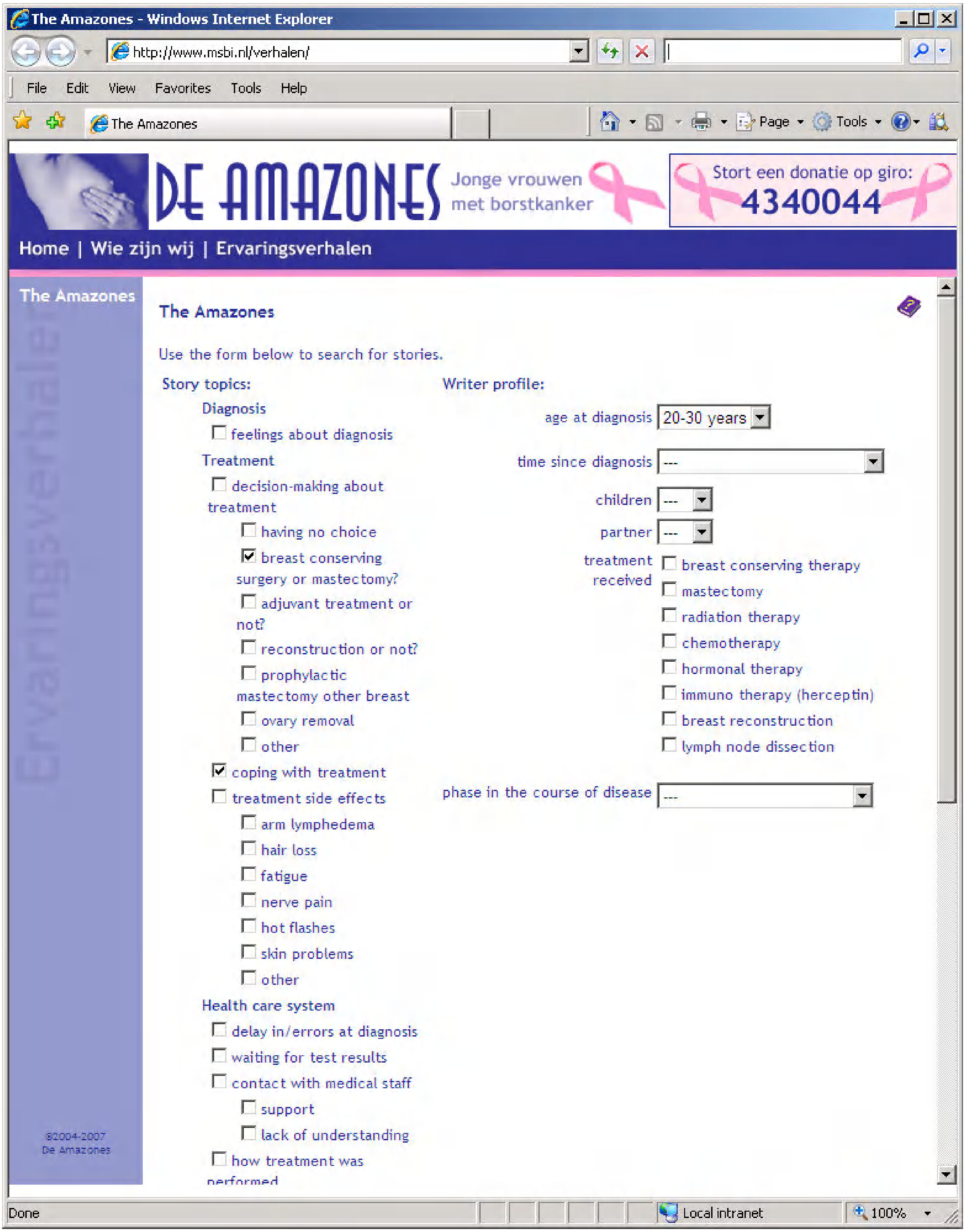

Screenshot 4b: A list of stories retrieved in the combination group.

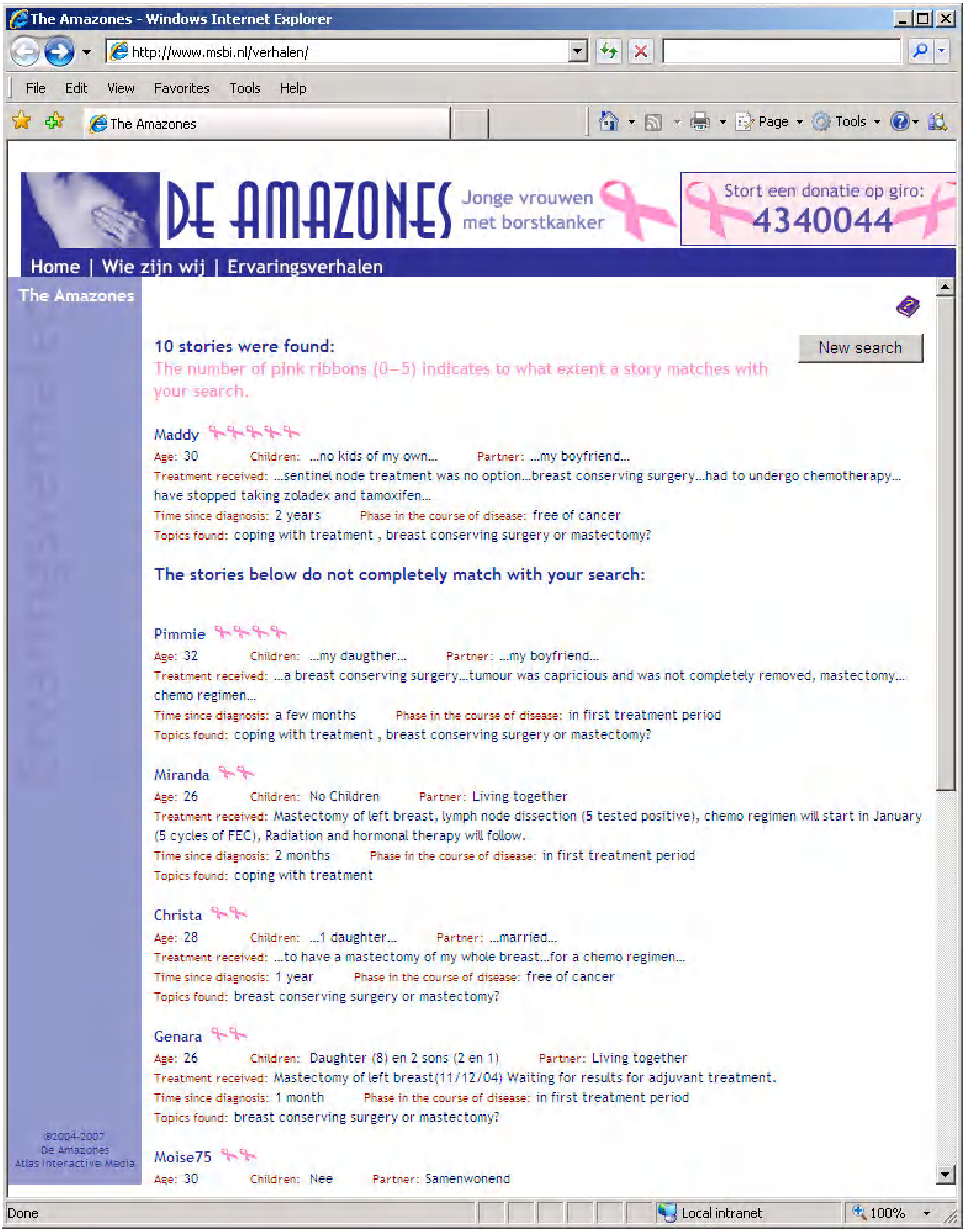

Supplement: Supplementary file 2 [file jmir_v12i1e7_app2.pdf]
