# Supplementary material for: How Breast Cancer Patients Want to Search for and Retrieve Information From Stories of Other Patients on the Internet: an Online Randomized Controlled Experiment
Source: J Med Internet Res. 2010 Mar 9;12(1):e7. doi: 10.2196/jmir.1215 (PMC2855205; doi:10.2196/jmir.1215)
Supplement: Supplementary file 3 [file jmir_v12i1e7_app3.pdf]

Multimedia Appendix III:
An overview of the constructs and items belonging to the three main outcome measures.

Appendix to:
Overberg R, Otten W, De Man A, Toussaint P, Westenbrink J, Zwetsloot-Schonk B. How breast cancer patients want to search for and retrieve information from stories of other patients on the Internet: an online randomised controlled experiment.

Constructs: 1. ‘Satisfaction with the search process’

<u>1.a. Opinion about the search facility (Cronbach’s alpha = .88):</u>
Five 5-point semantic differentials:
- poor … good
- difficult … easy
- not at all helpful … very helpful
- unpleasant … pleasant
- not convenient … convenient

<u>1.b. Opinion about the number of search options (Cronbach’s alpha = NA):</u>
Response categories: ‘too few’, ‘quite few’, ‘not few, not many’, ‘quite many’, ‘too many’
- What is your opinion on the number of search options?

(For analysis, this item was recoded into 3 points 1=negative opinion (‘too few’ or ‘too many’), 2=slightly negative opinion (‘quite few’ or ‘quite many’), and 3= positive opinion (‘not few, not many’)

<u>1.c. The extent to which the search options enable finding the information one was looking for (Cronbach’s alpha = .75):</u>
Response categories: ‘disagree’, ‘disagree a bit’, ‘neither disagree nor agree’, ‘agree a bit’, ‘agree’.
The ways in which I could search…
- …did *not* enable me to find what I wanted to know.
- …did enable me to find information that is important to me.
- …did *not* enable me to find stories that were helpful to me.
- …did enable me to find what I was searching for.

<u>1.d. Recommendation to others’ and future own use (Cronbach’s alpha = .82):</u>
Response categories: ‘yes’, ‘probably yes’, ‘maybe yes, maybe not’, ‘probably not’, ‘no’.
- Would you recommend others to search for stories in this way?
- Would you yourself search more often for stories in this way?

<u>1.e. Overall satisfaction with the search facility (Cronbach’s alpha = NA):</u>
One 10-point semantic differential:
- very poor … excellent

Constructs: 2. ‘Satisfaction with the stories retrieved’

2.a. Opinion about the stories retrieved (Cronbach’s alpha = .71):
Six 5-point semantic differentials:
- poor … good
- difficult … easy
- not at all helpful … very helpful
- not informative … informative
- frightening … not frightening
- I already knew everything … everything was knew to me

2.b. Opinion about the number of stories retrieved (Cronbach’s alpha = NA):
Response categories: ‘too few’, ‘quite few’, ‘not few, not many’, ‘quite many’, ‘too many’.
- What is your opinion on the number of stories retrieved?

(For analysis, this item was recoded into 3 points 1=negative opinion (‘too few’ or ‘too many’), 2=slightly negative opinion (‘quite few’ or ‘quite many’), and 3= positive opinion (‘not few, not many’)

2.c. Opinion about the list of stories displayed after a search (Cronbach’s alpha = .76):
Response categories: ‘disagree’, ‘disagree a bit’, ‘neither disagree nor agree’, ‘agree a bit’, ‘agree’.
- It was clear to me which story from the list I had to click onto to find the information I was looking for.
- The list of retrieved stories displayed after a search, was *not* helpful to me.
- It was difficult for me to decide which story I should read first.
- The way in which the list of retrieved stories was displayed, was clear to me.

2.d. The extent to which the stories retrieved covered one’s information need (Cronbach’s alpha = .82):
Response categories: ‘disagree’, ‘disagree a bit’, ‘neither disagree nor agree’, ‘agree a bit’, ‘agree’.
- I have found the information I was looking for.
- I am *not* satisfied with the information I have found.
- The information satisfied my expectations.
- I did *not* find the information I wanted to know.

2.e. Recommendation to others’ and future own reading (Cronbach’s alpha = .77):
Response categories: ‘yes’, ‘probably yes’, ‘maybe yes, maybe not’, ‘probably not’, ‘no’.
- Would you recommend others to read these stories?
- Would you yourself read more of these kinds of stories?

2.f. Overall satisfaction with the stories retrieved (Cronbach’s alpha = NA):
One 10-point semantic differential:
- very poor … excellent

Constructs: 3. 'The stories' impact on coping with breast cancer'

3.a. The stories' impact on coping with breast cancer (Cronbach's alpha = .85):
Response categories: 'disagree', 'disagree a bit', 'neither disagree nor agree', 'agree a bit', 'agree'.
By reading the stories:

- …I have learnt things.
- …I view things differently.
- …I am able to understand my feelings better.
- …I see that certain emotions accompany learning to live with breast cancer.
- …I know what to do.
- …I see that others have experienced the same things.
